# Purine metabolism-related genes and immunization in thyroid eye disease were validated using bioinformatics and machine learning

**DOI:** 10.1038/s41598-023-45048-9

**Published:** 2023-10-26

**Authors:** Zixuan Wu, Yuan Gao, Liyuan Cao, Qinghua Peng, Xiaolei Yao

**Affiliations:** 1 Hunan University of Traditional Chinese Medicine, Changsha, 410208 Hunan Province China; 2 Department of Ophthalmology, the First Affiliated Hospital of Hunan University of Traditional Chinese Medicine, Changsha, 410007 Hunan Province China

**Keywords:** Computational biology and bioinformatics, Immunology, Biomarkers

## Abstract

Thyroid eye disease (TED), an autoimmune inflammatory disorder affecting the orbit, exhibits a range of clinical manifestations. While the disease presentation can vary, cases that adhere to a prototypical pattern typically commence with mild symptoms that subsequently escalate in severity before entering a phase of stabilization. Notably, the metabolic activity of cells implicated in the disease substantially deviates from that of healthy cells, with purine metabolism representing a critical facet of cellular material metabolism by supplying components essential for DNA and RNA synthesis. Nevertheless, the precise involvement of Purine Metabolism Genes (PMGs) in the defensive mechanism against TED remains largely unexplored. The present study employed a bioinformatics approach to identify and validate potential PMGs associated with TED. A curated set of 65 candidate PMGs was utilized to uncover novel PMGs through a combination of differential expression analysis and a PMG dataset. Furthermore, GSEA and GSVA were employed to explore the biological functions and pathways associated with the newly identified PMGs. Subsequently, the Lasso regression and SVM-RFE algorithms were applied to identify hub genes and assess the diagnostic efficacy of the top 10 PMGs in distinguishing TED. Additionally, the relationship between hub PMGs and clinical characteristics was investigated. Finally, the expression levels of the identified ten PMGs were validated using the GSE58331 and GSE105149 datasets. This study revealed ten PMGs related with TED. PRPS2, PFAS, ATIC, NT5C1A, POLR2E, POLR2F, POLR3B, PDE3A, ADSS, and NTPCR are among the PMGs. The biological function investigation revealed their participation in processes such as RNA splicing, purine-containing chemical metabolism, and purine nucleotide metabolism. Furthermore, the diagnostic performance of the 10 PMGs in differentiating TED was encouraging. This study was effective in identifying ten PMGs linked to TED. These findings provide light on potential new biomarkers for TED and open up possibilities for tracking disease development.

## Introduction

Thyroid eye disease (TED), commonly known as Graves' ophthalmopathy or Graves' orbitopathy, is an inflammatory condition characterized by ocular tissue involvement. This disease is distinguished by lymphocyte infiltration, an increase in orbital fat, and edema of the extraocular muscles^1,2^. TED is frequently connected with Graves' disease, a systemic autoimmune ailment marked by endocrine symptoms that have a major impact on afflicted persons' quality of life^3^. TED is dangerous since it can cause visual impairment, functional incapacity, and deformity. While TED is most commonly seen in people with Graves' hyperthyroidism, it can also appear in hypothyroid or euthyroid patients^4^. TED usually appears 18 months after the beginning of endocrine symptoms, with roughly 80% of patients having contemporaneous signs. While TED and Graves' hyperthyroidism can affect people of various ages, it is more common in women between the ages of 30 and 50. TED is expected to affect around 16 cases per 100,000 women and three cases per 100,000 men per year^5,6^. Cigarette smoking, a lengthy history of Graves' hyperthyroidism, inadequate thyroid dysfunction care, and past radioactive iodine therapy have all been recognized as risk factors^7^. TED is frequently diagnosed by a thorough medical history and physical examination. Ophthalmic symptoms are reported in up to 50% of Graves' hyperthyroidism patients^8^. Therefore, understanding the underlying molecular mechanisms implicated in TED is critical for developing innovative treatment options that might successfully prevent disease recurrence and improve patient outcomes.

Nutrient intake and metabolism are critical processes for all living creatures' existence. Metabolic reprogramming is important in tumor cell growth and survival in the context of cancer. Recent research has shown that oncogenic transition causes a different metabolic profile in tumor cells, resulting in changes in the tumor microenvironment (TME). The TME is a complex environment composed of several cell types buried inside a complicated matrix, characterized by insufficient oxygen and nutrition availability due to damaged or poorly established vasculature^9^. The study of non-tumor immune infiltration is becoming increasingly important as research advances. The immune response is inextricably connected to substantial changes in tissue metabolism, such as nutritional depletion, increased oxygen demand, and the production of reactive nitrogen and oxygen species^10^. Furthermore, different microenvironmental variables dramatically impact immune cell proliferation and functioning, implying that metabolic treatments have the potential to improve the efficacy of immunotherapies^11^.

Nutrient intake and metabolism are critical processes for all living creatures' existence. Purines are important metabolic products because they serve as building blocks for DNA and RNA, which are required for life to exist. Purines are also essential components of several biomolecules, including ATP, GTP, cAMP, NADH, and coenzyme A^12^. These chemicals participate in a variety of biological functions, including energy generation, signaling pathways, redox metabolism, and fatty acid synthesis. Purines are also important in immune responses and interactions between hosts and pathogens, including tumor cells^13^. Purine metabolism in mammalian cells is divided into two major pathways: de novo synthesis and the salvage route, which works in tandem with the former. The salvage pathway, which is in charge of recycling damaged purine bases, meets the vast majority of cellular purine needs. However, the requirement for purines increases considerably in rapidly proliferating cells and tumor cells, resulting in activation of the de novo synthesis pathway to fulfill their needs^14^.

Purines, in particular, play an important function in tumor cell reproduction. As a result, purine antimetabolites were created as the first generation of anticancer medications and are now used to treat patients with acute lymphocytic leukemia, acute myeloid leukemia, and chronic myeloid leukemia^15^. While researchers' attention has shifted to non-neoplastic disorders, this work adds to the body of data supporting the use of purine antimetabolites in disease therapy. These medicines work by slowing cell growth and inhibiting DNA synthesis. Purinosomes, cellular entities intimately connected to purine metabolism and controlled by the cell cycle, have just been discovered^16^. These findings have opened up new possibilities for cancer therapy techniques that target purinosome development and purine metabolism. Despite the intriguing promise of targeting Purine Metabolism in conjunction with immunotherapy, its immunogenicity and immunotherapy landscape remains substantially studied, particularly in the context of TED. As a result, the current study was designed to offer a complete overview of PMGs and immunotherapy in TED, with the goal of shedding light on this understudied field of research.

**Figure 1:**
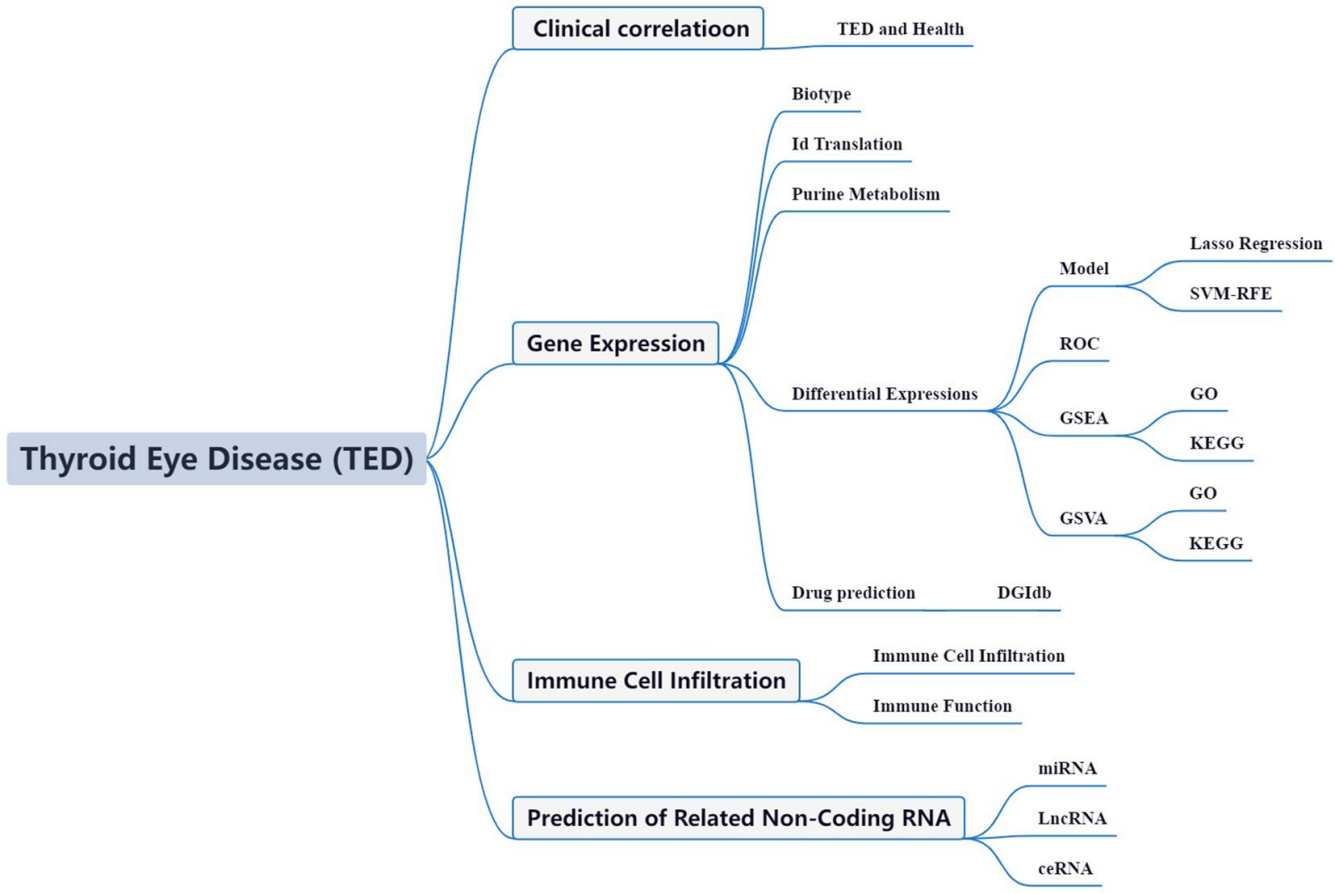
Framework. In an effort to advance our understanding of TED, patient-derived datasets were sourced from the GEO repositories. Specifically, the GSE58331 dataset served as the primary cohort, whereas the GSE105149 dataset was employed for validation purposes. Through rigorous matching of PMGs, we conducted differential expression analyses and subsequent risk model generation. Our analytical framework revealed a subset of PMGs with prognostic implications in TED, substantiating their potential as biomarkers. To further elucidate the functional roles of these genes, we executed a comprehensive set of bioinformatics analyses encompassing GO, KEGG, and GSEA. These analyses were facilitated through integration with multiple databases, providing a multi-dimensional view of the implicated PMGs in the context of cellular processes, signaling pathways, and gene networks. Lastly, to explore the immune landscape and gain insights into the transcriptional alterations, we performed a detailed assessment of immune cell infiltration, functional modulations, and RNA-level changes. This multifaceted approach offers a robust framework for understanding the implications of PMGs in TED, paving the way for future targeted interventions.

The TED Initiative's availability of high-throughput transcriptome sequencing data and complete clinical annotations has provided researchers with an unprecedented chance to examine the transcriptional alterations and related molecular pathways implicated in TED^17,18^. Bioinformatics analysis of these datasets produced surprising results, offering new insights into the underlying pathophysiology and processes of TED from a variety of viewpoints. However, no study to date has specifically employed bioinformatics approaches to assess the potential relevance of PMGs in TED. Consequently, the primary objective of this study was to explore the utilization of PMGs in the context of TED using TED-related GEO datasets. By leveraging these valuable resources, we aim to enhance our understanding of the involvement of PMGs in TED and shed light on their potential implications for disease mechanisms and therapeutic strategies (Fig. 1).

## Materials and methods

The methodologies proposed by Zi-Xuan Wu et al. in 2023 were employed in this study^19^.

### Raw data

The GEO datasets GSE58331 and GSE105149 were utilized in this study. The platform used was GPL570-55,999. GSE58331 served as the training group, while GSE105149 served as the test group. A total of 175 PMGs were included from the MSigDB (Table S1).

### Analysis of differentially expressed genes (DEGs)

Transcriptomic data underwent meticulous curation and normalization via Perl scripting, followed by conversion of identifiers to their respective gene nomenclatures. A comparative analysis between TED and normal tissue revealed differentially expressed PyMGs. After standardizing the data from GSE58331, differential expression analysis was performed using criteria of FDR < 0.05 and |log2FC|≥ 1 to identify DEGs among the PMGs. The functional implications of these DEGs were further examined. Then, Pearson's correlation coefficient was employed to analyze the statistically significant and highly correlated genes within modules using the correlation analysis provided by the corrplot package.

### GO and KEGG analysis

The biological functions and pathways associated with the identified DEGs were explored using GO and KEGG analysis. Specifically, R was utilized to investigate the impact of differentially expressed PMGs on Biological processes (BP), molecular functions (MF), and cellular components (CC).

### Model construction and analysis of immune cell infiltration

For model construction, the glmnet package was employed for Lasso regression analysis along with cross-validation. Additionally, the support vector machine recursive feature elimination algorithm (SVM-RFE) was utilized to build a machine learning model using the e1071 package. Cross-validation was used to assess the model's error and accuracy. Furthermore, Lasso and SVM algorithms were used to construct a model to rank the significance of feature genes. Immune cell composition was analyzed using the CIBERSORT method^20^.

SVM-RFE represents a sequential backward selection algorithm predicated on the maximum margin principle inherent to SVMs. In its inaugural iteration, the algorithm trains an optimized SVM model utilizing the full feature set available in the given dataset. Subsequent to this, each feature is scored, and these scores are ordered in a descending manner. The feature associated with the lowest score is then identified and eliminated from the dataset. This iterative process is perpetuated until a solitary feature remains, culminating in a refined feature subset optimized for model performance. By operationalizing this approach, SVM-RFE effectively navigates the high-dimensional feature space, mitigating the risk of overfitting while honing in on the most salient features. Such a procedure serves as a robust strategy for feature selection, particularly in complex datasets where discerning the most informative features is non-trivial. The methodology thus facilitates enhanced generalizability and predictive accuracy in machine learning models, making it particularly applicable to bioinformatics, finance, and other domains requiring intricate feature selection mechanisms.

### GSEA and GSVA

To identify relevant functions and pathway alterations across multiple samples, we employed GSEA^21^ and GSVA^22^. These computational tools allowed us to assess the dynamic activities and pathway changes within different risk subcategories by analyzing associated scores and visualizations. Furthermore, we utilized R to investigate the impact of differentially expressed PMGs on BP, MF, and CC, and pathways.

### Drug-gene interactions

As the field of bioinformatics progresses, the identification of potential biomarkers has become increasingly important for the development of biological models and effective diagnostic strategies in various diseases. However, it is crucial to understand how to effectively translate these biomarkers into clinical applications. Therefore, accurate prediction of drug responses based on informative markers is paramount for future prevention and treatment strategies in TED. Validated biomarkers serve as crucial reference points for targeted therapies. In this study, we utilized the DGIdb database (https://dgidb.genome.wustl.edu/) to predict drug interactions with the identified hub genes, enabling us to explore potential therapeutic interventions for TED.

### Identification of common miRNAs and lncRNAs

Non-coding RNA transcripts, including microRNAs (miRNAs) and long non-coding RNAs (lncRNAs), play pivotal roles in genetic regulation. MiRNAs can modulate gene expression by either enhancing mRNA degradation or inhibiting translation. On the other hand, lncRNAs are non-coding RNA molecules typically composed of approximately 200 nucleotides. They regulate various physiological and biochemical cellular processes by mediating chromosomal changes, transcriptional activation, and interference. Recent studies have highlighted extensive crosstalk between miRNAs and lncRNAs, involving competition for binding between miRNAs, lncRNAs, and other regulatory targets. Notably, certain competitive endogenous RNAs (ceRNAs) have been identified, where an lncRNA functions by sequestering miRNAs. Thus, in this study, we aim to investigate whether specific miRNAs and lncRNAs exhibit shared regulatory mechanisms and participate in developmental processes relevant to TED.

### Construction of a network of common mRNA-miRNA-lncRNA genes

To obtain information regarding target genes for the identified common miRNAs and lncRNAs, we utilized empirically validated databases, including miRTarbase and PrognoScan, which provide comprehensive miRNA-lncRNA-target relationships. By intersecting the target genes of the common mRNA-miRNAs-lncRNA and the shared genes identified in TED, we constructed a regulatory network. The network was visualized using Cytoscape software, allowing for a comprehensive understanding of the intricate interactions between mRNA, miRNA, and lncRNA genes involved in TED.

### Ethics approval and consent to participation

This manuscript is not a clinical trial, hence the ethics approval and consent to participation are not applicable.

## Results

### Identification of DEGs and principal component analysis

**Figure 2:**
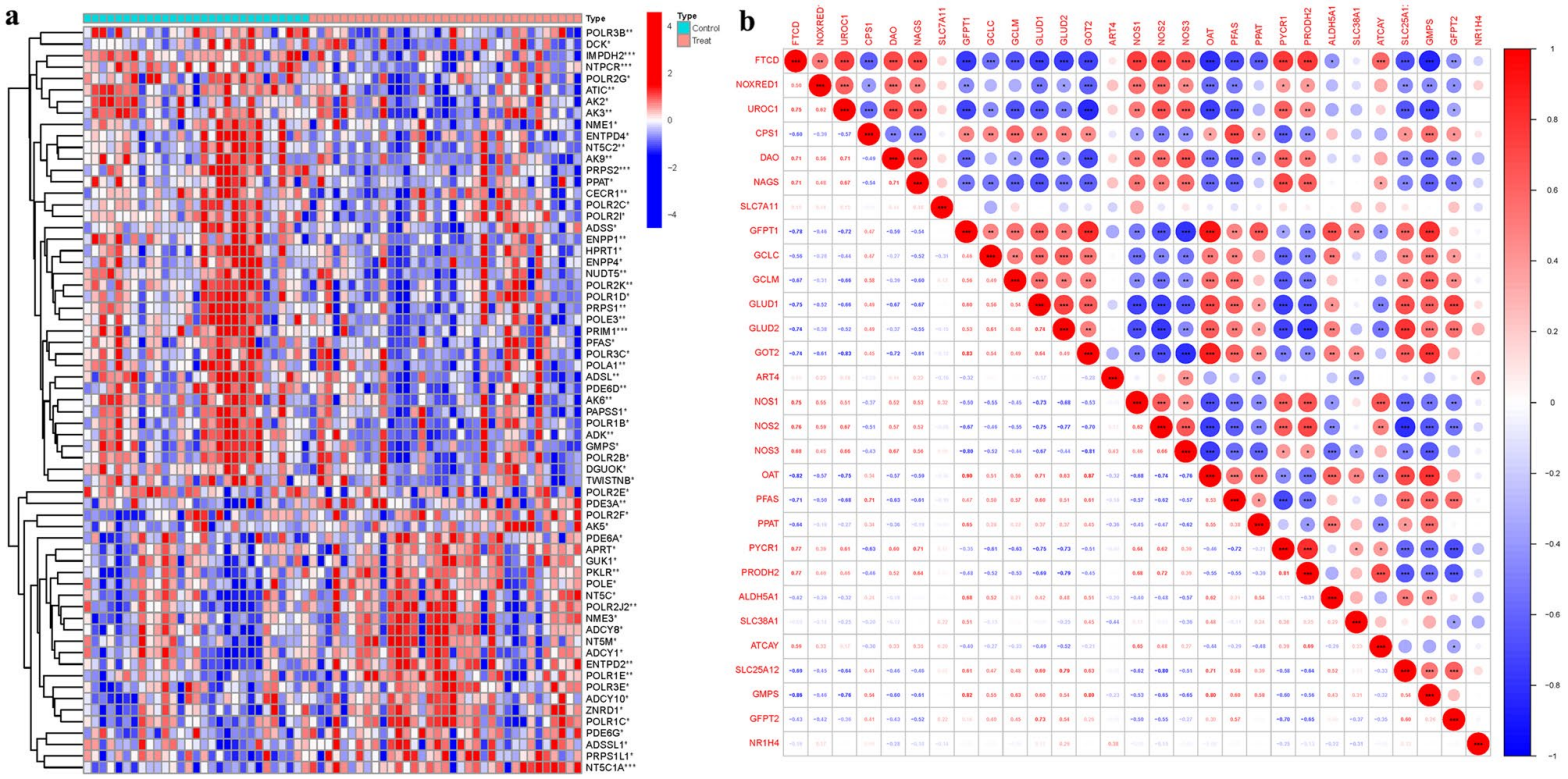
Principal Component Analysis. (**a**) Analysis of difference. (**b**) Analysis of correlation. In a meticulous evaluation of gene expression profiles, a marked divergence was observed between treatment and control cohorts. Within the treatment group, PMGs manifested notable variances, encompassing genes such as PDE3A, POLR2F, AK5, PDE6A, APRT, GUK1, PKLR, POLE, NT5C, POLR2J2, and NME3. Conversely, the control group displayed distinct expression profiles for genes including POLR3B, DCK, IMPDH2, NTPCR, POLR2G, ATIC, AK2, AK3, NME1, and ENTPD4.

Among the 65 examined PMGs, several exhibited significant differences in their expression levels. Gene clustering analysis revealed distinct clusters between the treatment and control groups. Noteworthy PMGs in the treatment group included PDE3A, POLR2F, AK5, PDE6A, APRT, GUK1, PKLR, POLE, NT5C, POLR2J2, and NME3, while the control group included POLR3B, DCK, IMPDH2, NTPCR, POLR2G, ATIC, AK2, AK3, NME1, ENTPD4, among others (Fig. 2a). Correlation analysis was conducted among these PMGs, and a correlation matrix was generated for visualization (Fig. 2b) (Table S2).

### Enrichment analysis of PMGs

**Figure 3:**
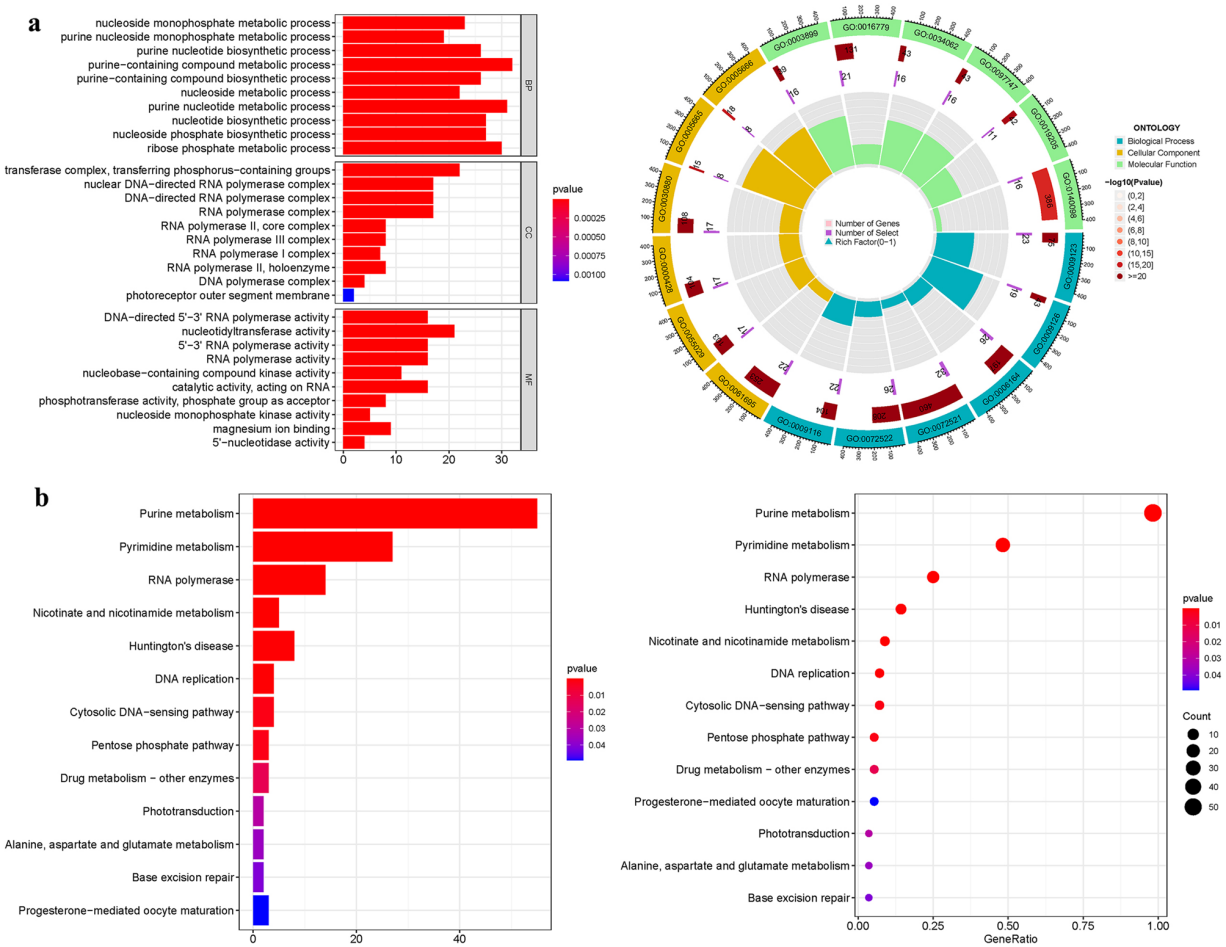
For PMGs, GO, and KEGG analyses were performed. (**a**): The GO circle illustrates the scatter map of the selected gene's logFC. (**b**): The KEGG barplot and bubble illustrates the scatter map of the logFC of the indicated gene. Through GO enrichment analysis, we identified an ensemble of 351 core target genes that are intricately implicated across an array of BP, MF, and CC. This comprehensive gene set offers a profound insight into the multidimensional landscape of cellular functionality. Building upon this, KEGG enrichment analysis further demarcated the principal signaling pathways. Remarkably, we discovered that the over-expressed genes predominantly converge upon metabolic pathways.

GO enrichment analysis identified 351 core target genes involved in various BP, MF and CC. In the MF category, guanyl nucleotide binding (GO:0,019,001), guanyl ribonucleotide binding (GO:0,032,561), and nucleoside binding (GO:0,001,882) were prominent. The CC category was primarily associated with the basal part of the cell (GO:0,045,178), transferase complex transferring phosphorus-containing groups (GO:0,061,695), and nuclear chromosome (GO:0,000,228). The BP category included RNA splicing (GO:0,008,380), purine-containing compound metabolic process (GO:0,072,521), and purine nucleotide metabolic process (GO:0,006,163). Additionally, Kyoto Encyclopedia of Genes and Genomes (KEGG) enrichment analysis revealed that upregulated genes were primarily involved in Pyrimidine metabolism (hsa00240), Progesterone-mediated oocyte maturation (hsa04914), Huntington's disease (hsa05016), and Purine metabolism (hsa00230) (Fig. 3 and Table S3a-b).

### Model construction

**Figure 4:**
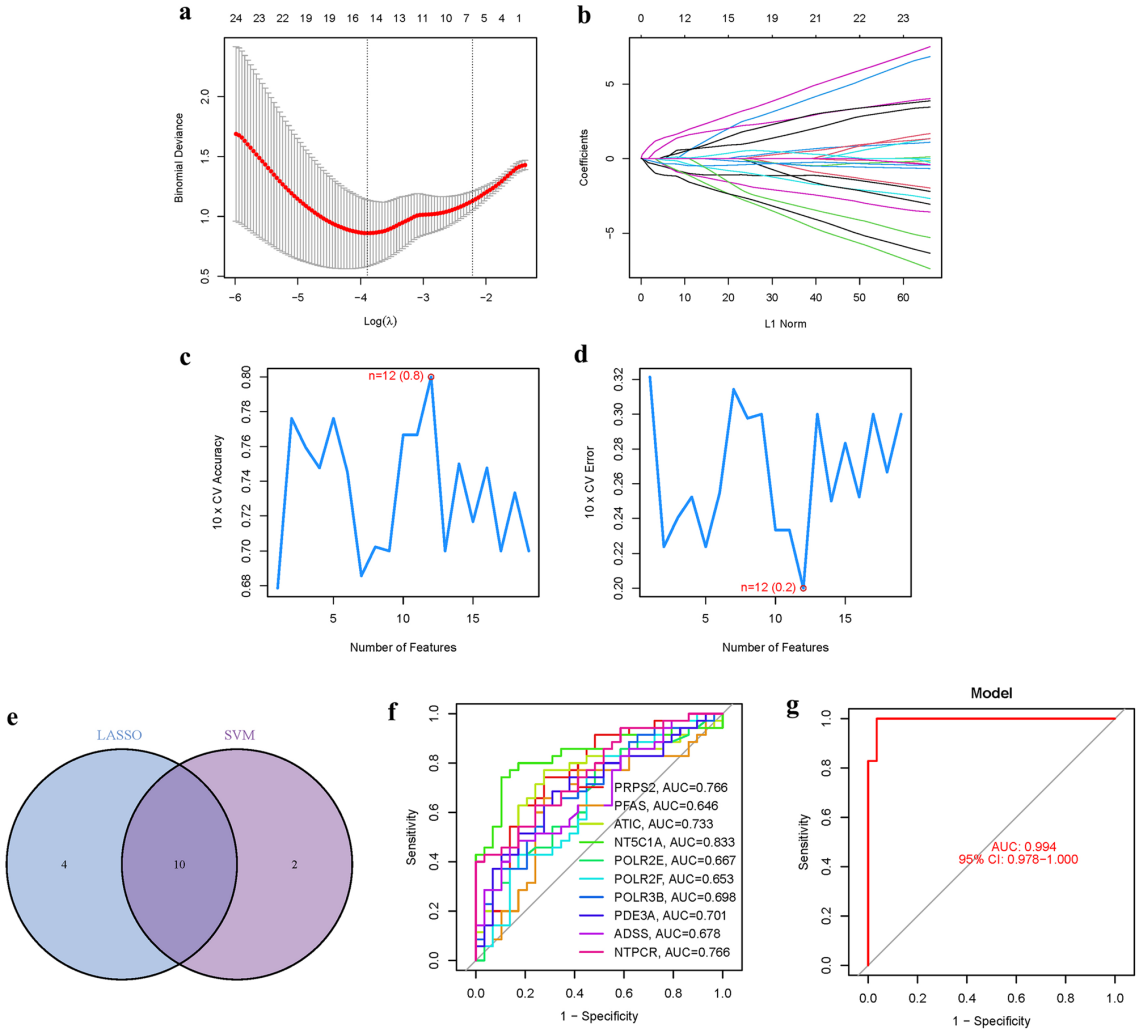
The development of the PMGs signature. (**a)**: Regression of the 10 TED-related genes using LASSO, It can be seen that these 10 genes are significantly related to TED and PMGs. (**b**): Cross-validation is used in the LASSO regression to fine-tune parameter selection, Cross-validation indicated that the results obtained by the proposed algorithm were stable. (**c**-**d**): Accuracy and error of this model, These results suggest that the stability of this study is good. (**e**): Venn, The intersection core genes of lasso and SVM-RFE were 10. (**f**): AUC of 10 hub genes. (**g**): AUC of train group.

A gene signature was established using LASSO and Cox regression analysis with optimized value selection (Fig. 4a-b). To validate the accuracy and reliability of the model, a machine learning model was built using SVM-RFE. The model demonstrated an accuracy of 0.8 and an error rate of 0.2 (Fig. 4c-d). The intersection of the ten PMGs identified by LASSO and SVM exhibited strong concordance (Fig. 4e). Comparing the model with the 10 hub genes, high accuracy readings were observed for all of them: PRPS2 (AUC = 0.766), PFAS (AUC = 0.646), ATIC (AUC = 0.733), NT5C1A (AUC = 0.833), POLR2E (AUC = 0.667), POLR2F (AUC = 0.653), POLR3B (AUC = 0.698), PDE3A (AUC = 0.701), ADSS (AUC = 0.678), NTPCR (AUC = 0.766) (Fig. 4f). In GSE58331, an Area Under the Curve (AUC) of 0.994 (95% CI 0.978–1.000) was achieved, indicating that the prediction model is highly accurate and robust (Fig. 4g) (Table S4).

### Model validation

**Figure 5:**
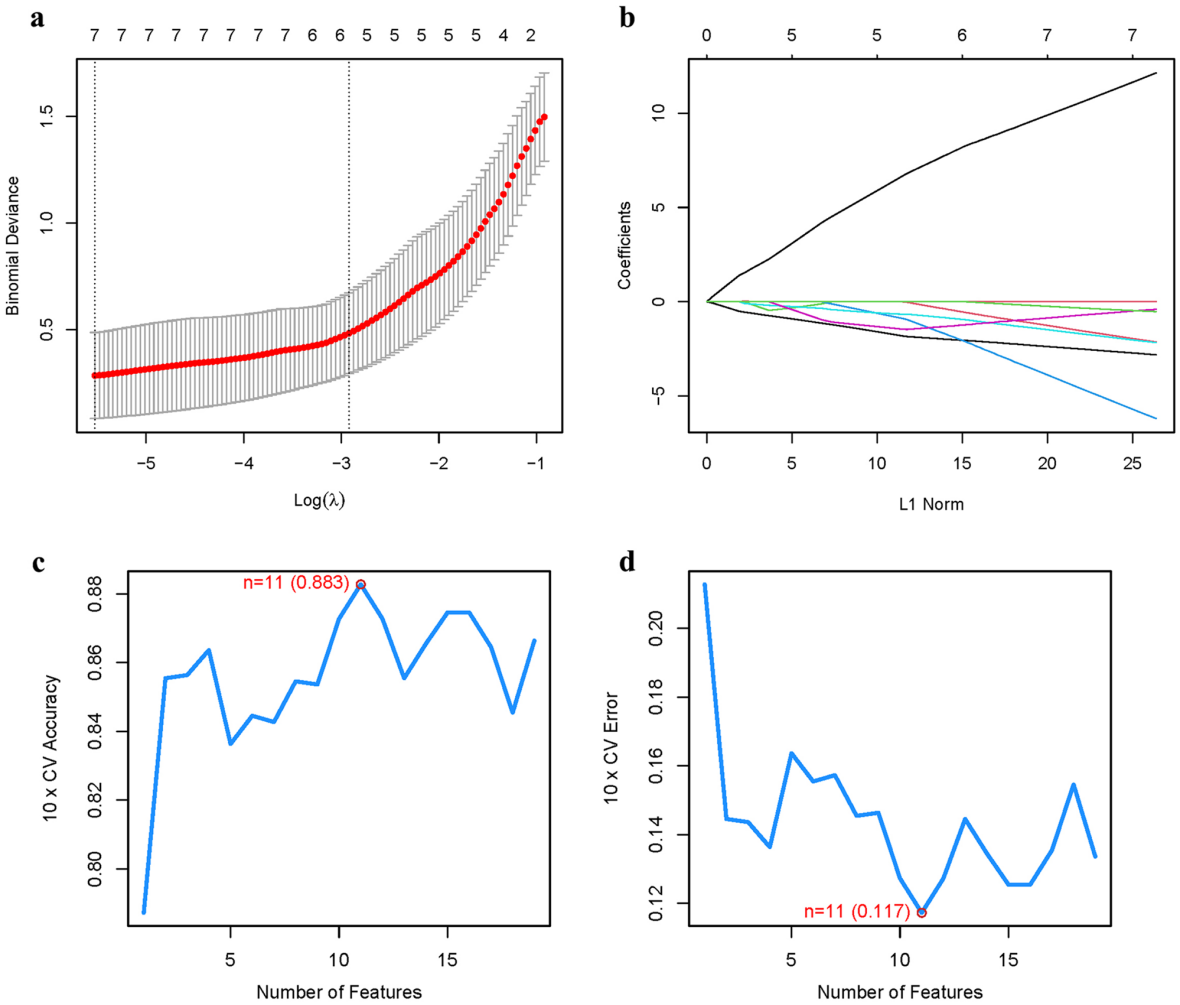
The validation of the PMGs signature. (**a**): Regression of the 10 TED-related genes using LASSO, It can be seen that these 10 genes are significantly related to TED and PMGs. (**b**): Cross-validation is used in the LASSO regression to fine-tune parameter selection, Cross-validation indicated that the results obtained by the proposed algorithm were stable. (**c**-**d**): Accuracy and error of validation model, These results suggest that the stability of this study is good.

In GSE58331, we used the same algorithm for validation. A gene signature was established using LASSO and Cox regression analysis with optimized value selection (Fig. 5a-b). To validate the accuracy and reliability of the model, a machine learning model was built using SVM-RFE. The model demonstrated an accuracy of 0.883 and an error rate of 0.117 (Fig. 5c-d).

### Gene set enrichment analysis

**Figure 6:**
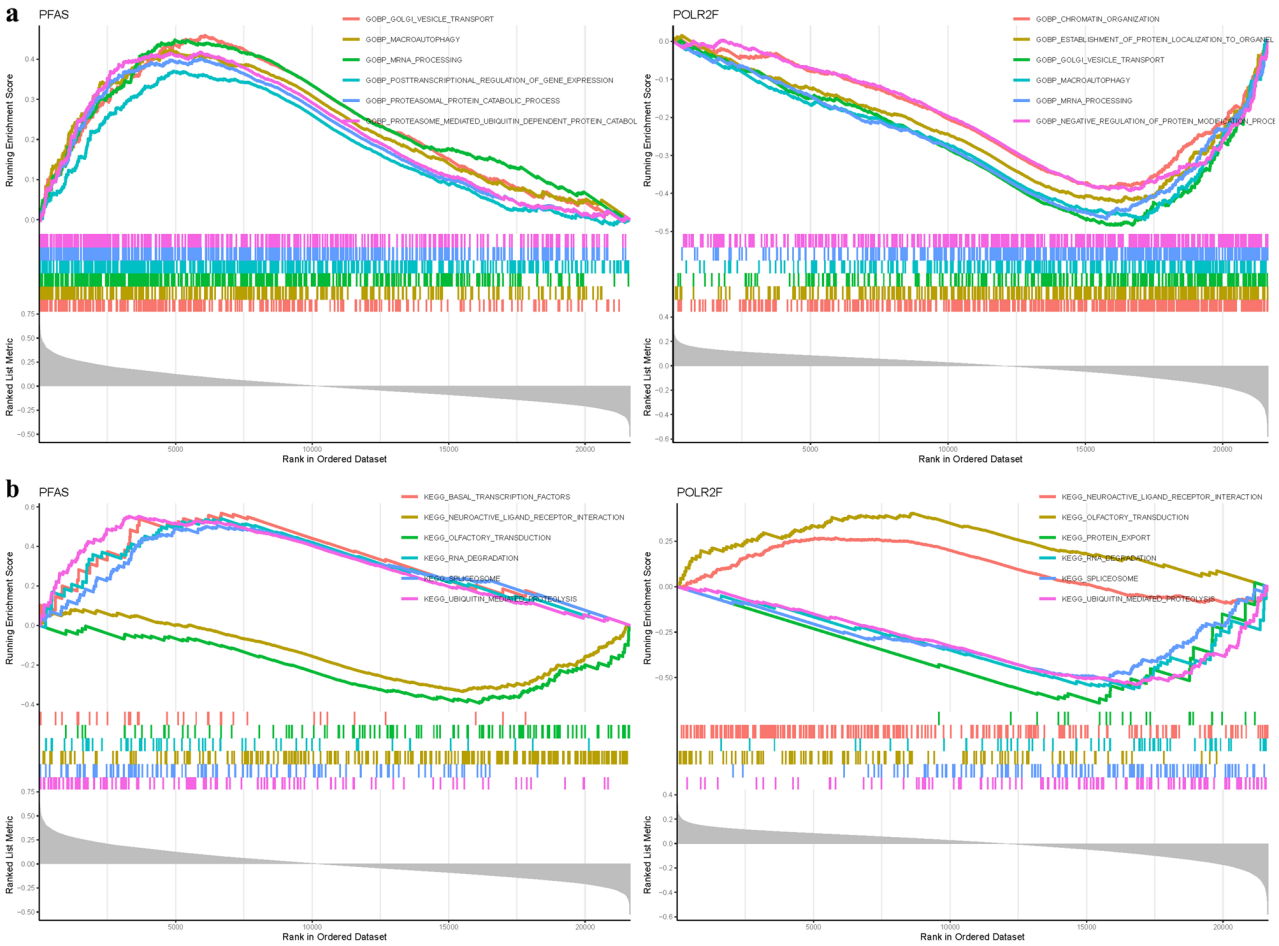
GSEA of Analysis in PFAS and POLR2F. (**a**): GO. (**b**): KEGG.

Through literature evaluation and analysis of hub gene sensitivity within the model, it was determined that PFAS and POLR2F may be the most relevant genes to TED. In terms of GO analysis, PFAS was found to be associated with Biological Processes such as golgi vesicle transport, mRNA processing, and proteasome-mediated ubiquitin-dependent protein catabolism. On the other hand, POLR2F was primarily involved in golgi vesicle transport, macroautophagy, and mRNA processing (Fig. 6a). In KEGG analysis, PFAS was mainly associated with basal transcription factors, ubiquitin-mediated proteolysis, and RNA degradation, while POLR2F was involved in neuroactive ligand-receptor interaction, olfactory transduction, and ubiquitin-mediated proteolysis (Fig. 6b) (Table S5).

### Analysis of immune cells

**Figure 7:**
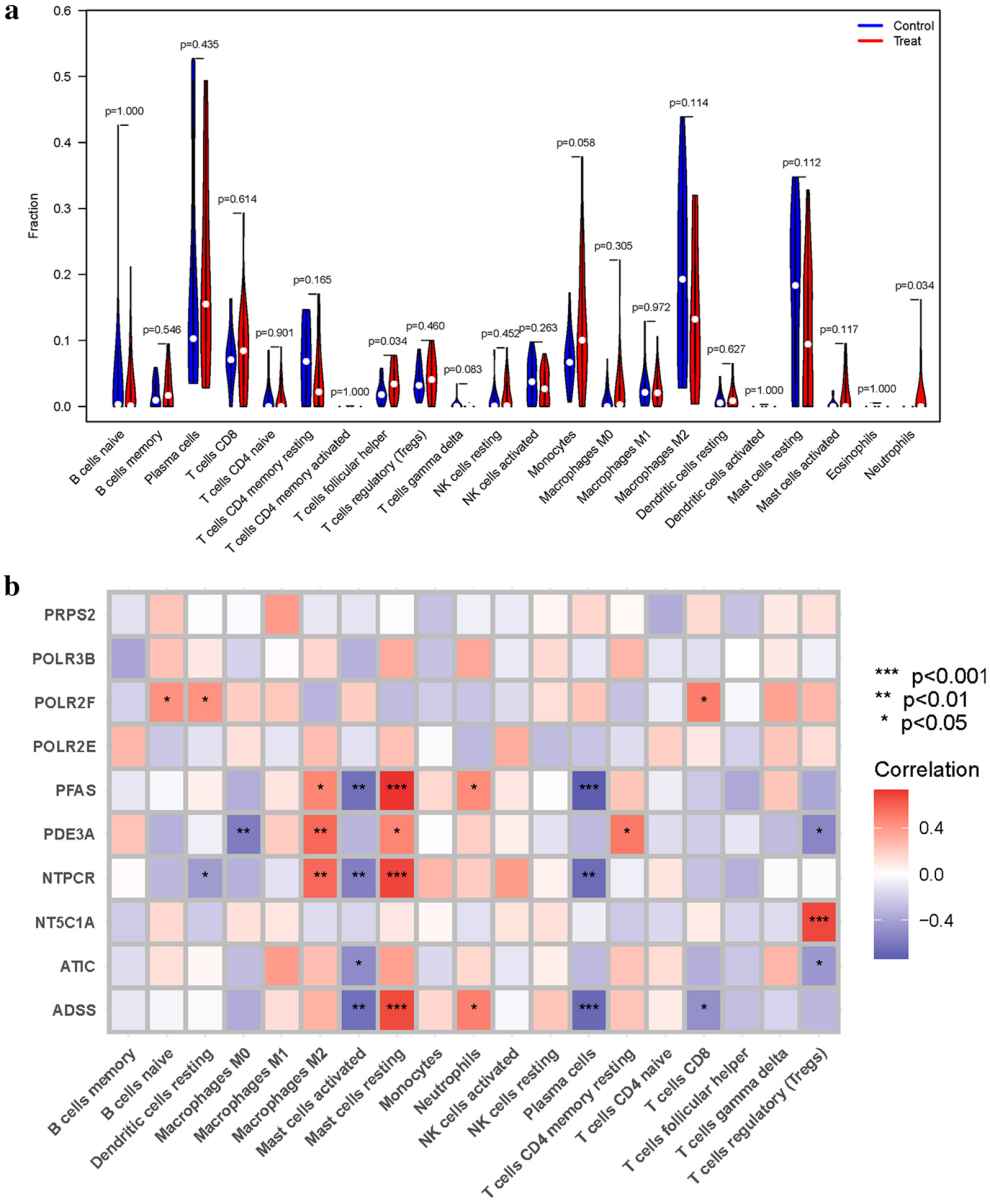
Expression of Immune cells. (**a**) Expression of immune cells in different clusters. (**b**) Correlation between PMGs and immune cells.

The immune microenvironment plays a pivotal role in the initiation and progression of TED. Expression patterns of T cells follicular helper and Neutrophils, which were highly expressed in the treatment group, were displayed using a vioplot (Fig. 7a). Furthermore, a correlation analysis was conducted to investigate the relationship between these genes and immune cells (Fig. 7b).

### GSVA

**Figure 8:**
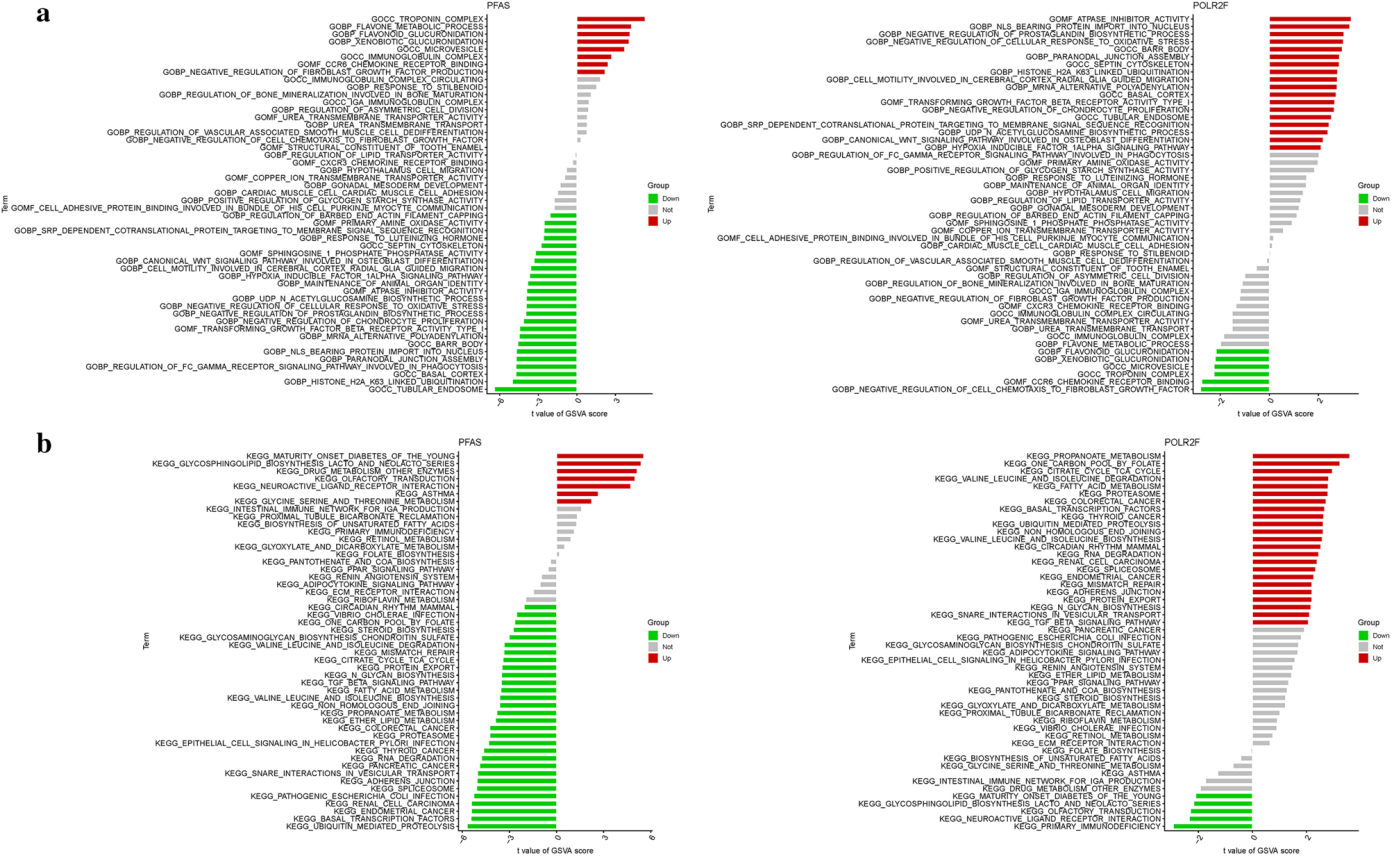
GSVA of Analysis in PFAS and POLR2F. (**a**): GO. (**b**): KEGG.

In the GO analysis, PFAS was primarily associated with BP such as flavonoid glucuronidation, flavone metabolic process, CC including troponin complex, microvesicle, and MF such as ccr6 chemokine receptor binding and urea transmembrane transporter activity. On the other hand, POLR2F was mainly involved in BP related to the negative regulation of prostaglandin biosynthetic process, nls bearing protein import into the nucleus, MF including atpase inhibitor activity and transforming growth factor beta receptor activity type I, and CC related to barr body and septin cytoskeleton (Fig. 8a). In terms of KEGG analysis, PFAS was predominantly associated with fatty acid metabolism, valine leucine and isoleucine degradation, citrate cycle (TCA cycle), one carbon pool by folate, and propanoate metabolism. POLR2F was involved in olfactory transduction, drug metabolism other enzymes, glycosphingolipid biosynthesis lacto and neolacto series, and maturity onset diabetes of the young (Fig. 8b).

### Drug-gene interactions

**Figure 9:**
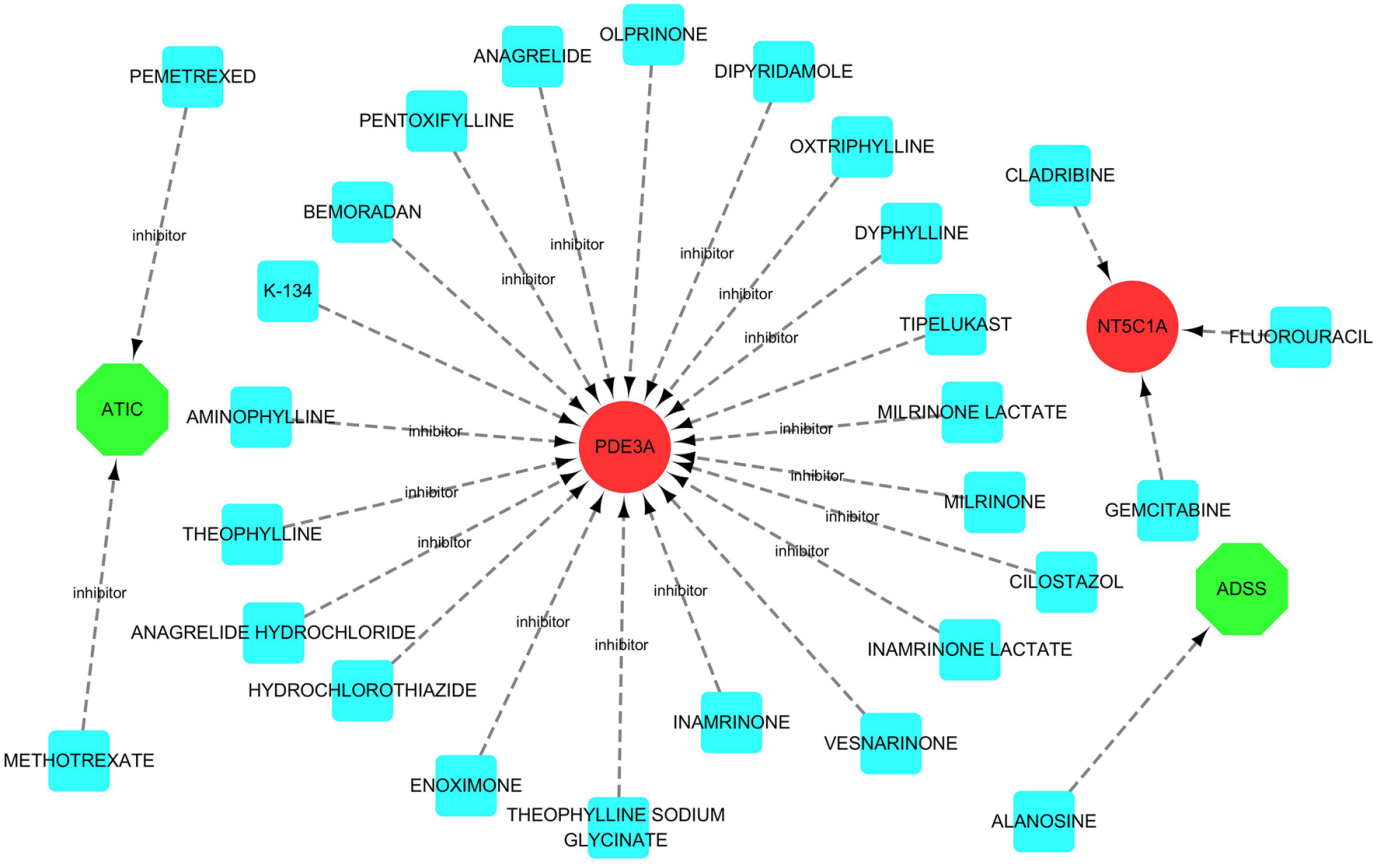
Drug-gene interactions. Note: Red circles are up-regulated genes, green hexagons are down-regulated genes, and blue squares are associated drugs.

Three drugs, including PEMETREXED, METHOTREXATE, GEMCITABINE, CLADRIBINE, FLUOROURACIL, were predicted to interact with the twenty-seven hub genes (Table S6). To visualize the drug-gene interactions, Cytoscape 3.7.1 was utilized (Fig. 9).

### Identification of common RNAs and construction of miRNA-lncRNA shared genes network

**Figure 10:**
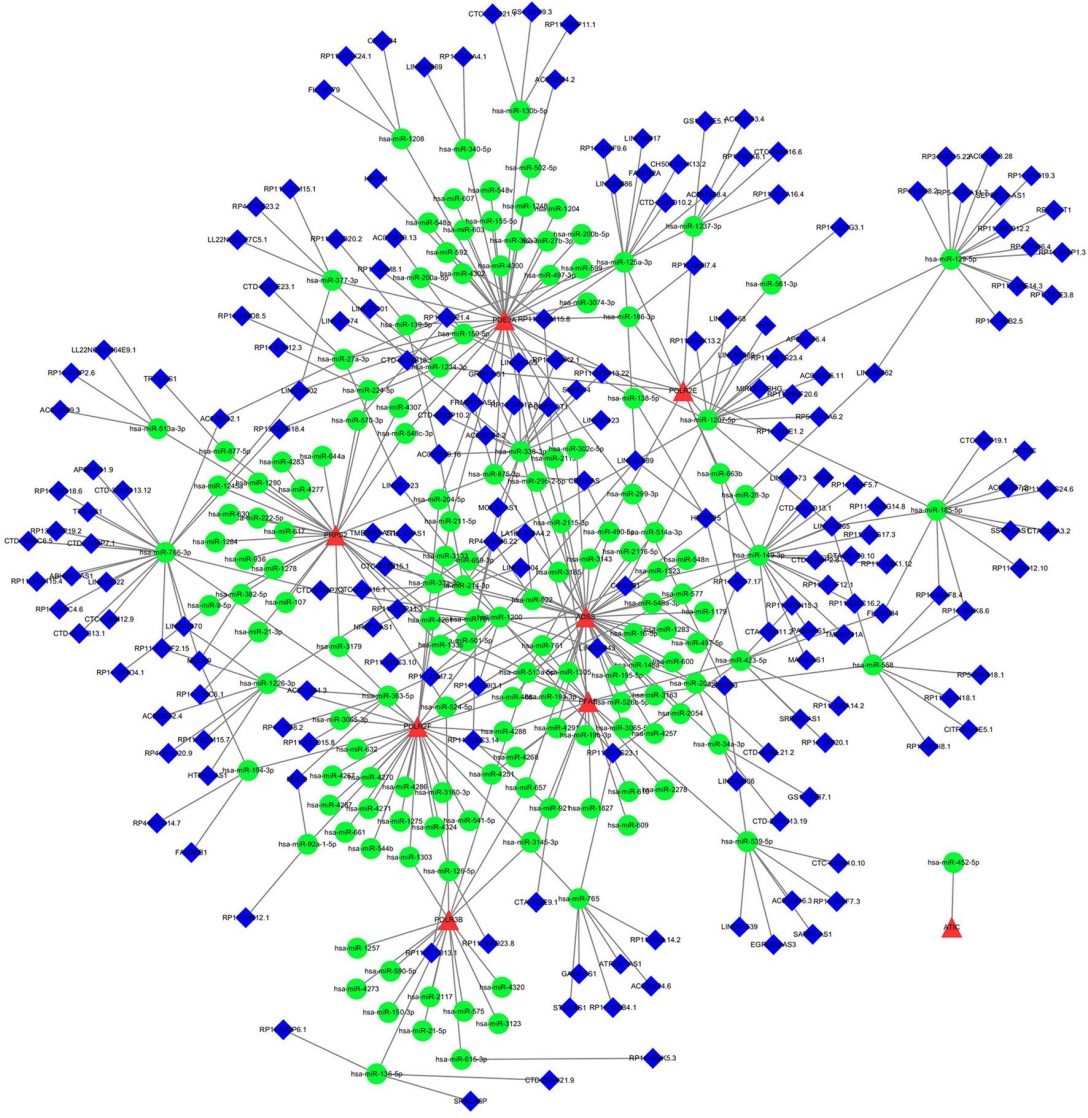
miRNAs-LncRNAs shared Genes Network. Note: Red circles are mrnas, blue quadrangles are miRNAs, and green triangles are lncRNAs.

A total of 192 miRNAs and 241 lncRNAs associated with TED were identified from three databases (Table S7a-b). The miRNA-lncRNA-gene network was constructed by intersecting these non-coding RNAs with the shared genes obtained through Lasso regression and SVM-RFE. The network consisted of 192 lncRNAs, 155 miRNAs, and several common genes, including the eight hub genes (ADSS, POLR2F, PDE3A, POLR2E, PRPS2, POLR3B, PFAS, and ATIC) (Fig. 10).

### Validation of hub genes

**Figure 11:**
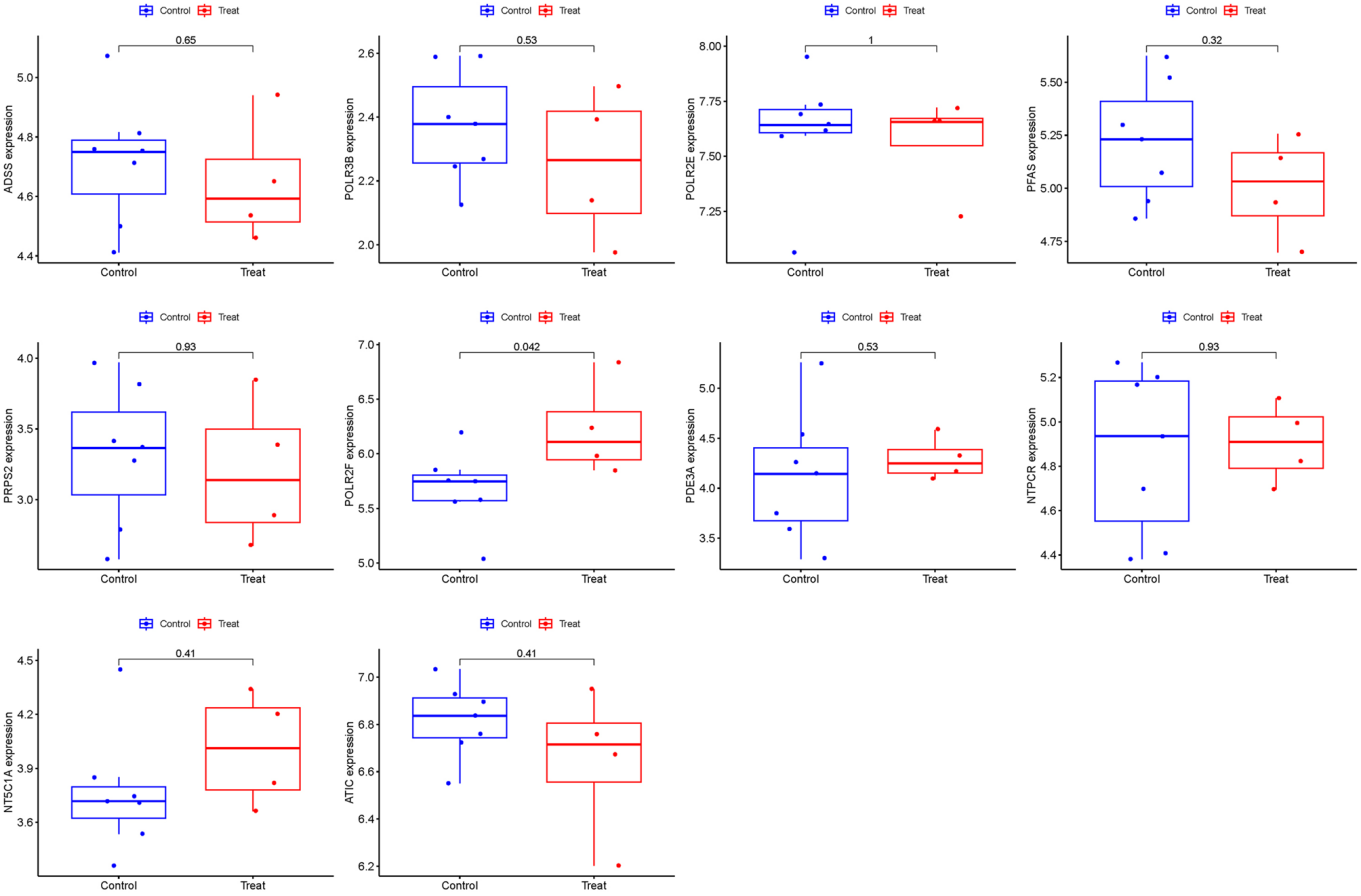
Ten hub genes were validated.

To further strengthen the confidence and prediction accuracy of the model, the GSE105149 dataset was utilized for validation. Among the ten PMGs, PFAS and POLR2F exhibited significant differences in the GSE105149 analysis, providing additional confirmation of their potential relevance to TED (Fig. 11).

### Discussions

TED is an uncommon inflammatory orbital disorder that can cause vision loss, facial deformities, and, in severe cases, total vision loss^23^. Diplopia (double vision), dry eyes, and optic nerve degeneration are all ophthalmic symptoms of TED. Proptosis (eyeball protrusion), eyelid retraction, increased orbital fat, and edema are all possible soft tissue alterations^24^. The traditional view of TED is based on Francis Rundle's categorization, which distinguishes two separate stages: an active and progressing inflammatory phase lasting 1 to 3 years, followed by an inactive fibrotic phase^25^. Tobacco use and radioactive iodine (RAI) exposure are known risk factors for TED, with a synergistic impact found when both variables are present^26^. While there is no unique hereditary vulnerability to TED, epigenetic processes and genetic polymorphisms may play a role in its development. TED has been associated to polymorphisms in genes encoding human leukocyte antigen, cytotoxic T-lymphocyte antigen 4, interleukin 23 receptor, protein tyrosine thyroglobulin, and thyroid-stimulating hormone receptor, according to several studies^27^. The journey toward elucidating unambiguous genetic associations between TED development and its concomitant disease severity necessitates further intricate investigation. Consequently, leveraging the precise and scalable methodologies of bioinformatics, this study meticulously orchestrates model construction and computation, aiming to provision definitive research trajectories for impending basic and clinical inquiries, thereby envisioning a future where the enigma of TED is deconstructed into actionable therapeutic strategies and insightful clinical interventions^28^.

In this situation, gene expression regulation is likely to be critical. Purine, a heterocyclic bicyclic aromatic chemical, is involved in many aspects of metabolism and cell signaling. Numerous purine nucleotide enzymes, both anabolic and catabolic in nature, have been linked to tumor cell growth and treatment resistance^29^. Uric acid imbalances in antioxidant and pro-inflammatory characteristics have the capacity to trigger and sustain tumor development. Through changes in the signal transduction pathway, disrupted purine nucleotide metabolism can modify gene and protein expression, increasing cell malignant transformation, invasion, and metastasis^30^. Several risk factors for a variety of ocular illnesses have been found in recent years. However, without rigorous review and substantial replication, the majority of these discoveries remain primarily speculative^31^. The majority of research has concentrated on the role of individual purine metabolism regulators in cancer, leaving the combined contributions of many purine metabolism-related genes in other diseases relatively unclear^32^. Tumor research advances have changed the emphasis from investigating tumor components to getting a thorough grasp of non-cancerous biology. In the realm of autoimmune diseases, an unbridled aggression of the immune system commonly emerges as a pervasive sequela, ushering in a cascade of sustained inflammation and subsequently cultivating a hyper-inflammatory state that frequently paves the way for disease progression. This immune response, encapsulating a paradox by acting as a double-edged sword, consequently poses substantial challenges in formulating therapeutic modalities and strategies. Within this context, the present investigation pivots towards an intensive exploration of purine metabolic processes in the establishment of TED, a pursuit teeming with potential and promise given the critical role of purine metabolism in cellular activities and inflammatory conditions.

Within the investigative sphere of TED, this pivotal inquiry meticulously delineates 65 DEGs with pronounced correlations to Purine Metabolism, weaving a complex narrative of cellular, genetic, and metabolic interplay. Employing a synergistic, multimodal analytic framework, which seamlessly integrates DEGs, Lasso regression, and SVM-RFE, we discerned a constellation of 10 hub PMGs-specifically, PRPS2, PFAS, ATIC, NT5C1A, POLR2E, POLR2F, POLR3B, PDE3A, ADSS, and NTPCR. Validation through external datasets not only buttresses the diagnostic salience of these hub genes but also entwines them into the complex etiopathogenetic narrative of TED. While the strides achieved are indubitably significant, an acknowledgment of existing lacunae is imperative. Presently, the empirical evidence anchoring these hub genes to the precise orchestration of transcription factors entwined in Purine Metabolism remains notably scant. This evidentiary dearth accentuates an imperative for subsequent, more in-depth explorations. A meticulous perusal of prevailing literature further amplifies the cardinal roles of PFAS and POLR2F in TED pathogenesis, earmarking them as luminous beacons for ensuing research pursuits.

PFAS constitute a class of synthetic chemicals ubiquitously deployed across a diverse array of industrial applications and consumer goods. Their recalcitrant nature, characterized by marked resistance to environmental degradation, has culminated in their pervasive bioaccumulation across ecosystems^33^. Reputed as "forever chemicals" owing to their persistent resilience, PFAS have been incontrovertibly identified in an extensive range of biological tissues-ranging from blood, hepatic and renal tissues, to cardiac, muscular, and cerebral matrices-in multiple species^34^. The scientific literature elucidates two primary mechanistic pathways through which PFAS traverse into the cerebral compartment: firstly, by compromising the integrity of tight junctions, thereby facilitating the disassembly of the blood–brain barrier (BBB); and secondly, via the exploitation of specialized transporters strategically located at the BBB interface. Intriguingly, the proclivity of PFAS to gain cerebral access and subsequently accumulate is not monolithic but is contingent upon their structural and physicochemical attributes^35^. For example, short-chain PFAS manifest diminished efficiencies in crossing cerebral barriers compared to their long-chain counterparts. Biomonitoring endeavors and experimental designs probing PFAS exposure have unequivocally demonstrated their accrual within cerebral structures in both human and non-human biota. Anatomically, PFAS accumulation is conspicuously evident within specialized neural regions, including but not limited to, the brain stem, hippocampus, hypothalamus, pons/medulla, and thalamus^36^. Such spatial distribution of PFAS within the brain imposes a consequential toxicological burden on the central nervous system, reinforcing the imperative for further rigorous examination. Collectively, these findings serve as an edifying foundation for understanding the ingress and bioaccumulation patterns of PFAS, thereby delineating avenues for subsequent investigative scrutiny and potential therapeutic intervention strategies^37^.

The findings of Antonacopoulou's study shed light on the higher expression of POLR2F and PRNP in carcinomas compared to normal tissue samples, indicating a possible role in colorectal cancer^38^. Lin established a 6-RBP gene signature consisting of POLR2F, DYNC1H1, SMAD9, TRIM21, BRCA1, and ERI1 in another bioinformatics work, allowing for a complete assessment of glioma and ischemic stroke. The RBPS 6-RBP gene signature, in particular, revealed independent prognostic potential for overall survival. Surprisingly, the study discovered a relationship between SMAD9 overexpression and dementia, indicating a possible link between SMAD9 and cognitive abnormalities. Conversely, POLR2F downregulation appears to be linked to age-related hypoxic stress, highlighting its possible role in physiological responses to oxygen deprivation during the aging process^39^. In the specialized milieu of TED, PMGs such as PFAS and POLR2F have been intriguingly implicated, as discerned from the patient cohort under our investigative lens, thereby lending heightened credence and legitimacy to our findings. Corroborating with the insights gleaned from the GSE105149 study, which suggested that a Purine Metabolism-related signature could effectively serve as a prognostic predictor, our results intertwine with this existing knowledge matrix, though it is pivotal to underscore the fact that the specific gene alterations associated with Purine Metabolism have hitherto been relatively underexplored in the scientific literature. In summation, our investigations not only substantively augment the nascent corpus of knowledge within this specific domain but also bequeath crucial insights, delicately poised to catalyze forthcoming investigational ventures and therapeutic innovations. By doing so, we carve a pathway towards a more nuanced understanding and potential novel therapeutic interventions in the complex and intricate realm of TED.

The TME is a complex environment that includes not only malignant cells but also a wide range of non-malignant cellular elements, vascular networks, and extracellular matrix. Myriad immune cell subtypes play critical regulatory roles in this dynamic ecology. Cumulative data highlights the substantial impact of the complicated interplay between neoplastic cells and the many aspects of the TME, which frequently results in immunological evasion of the immunological illnesses, stimulating Immune disease proliferation, recurrence, and metastasis. Despite great advances in cancer immunotherapy, the treatment paradigm is riddled with difficulties that prevent its widespread effectiveness. As a result, discovering new treatment targets and predicting biomarkers is critical for improving and enhancing immunotherapeutic efficacies. In this setting, an in-depth examination of immune cell infiltration inside the TME is critical. A slew of clinical studies have been conducted in the last 6–8 years to investigate various immune modulators, owing to a better knowledge of the molecular pathways underlying the pathogenesis of TED^40^. It is commonly assumed that thyroid autoantibodies cause TED by eliciting a cross-reactive reaction against the thyrotropin receptor (TSHR) and the IGF-1R on ocular fibroblasts. This sets off a chain reaction of events involving B cell-mediated mechanisms, such as autoantigen detection on orbital fibroblasts, subsequent T cell activation, migration of T cells, macrophages, and mast cells to the orbit, and an increasing release of proinflammatory cytokines^41,42^. Consequently, orbital fibroblasts become activated, initiating a self-perpetuating cycle characterized by increased cytokine production, proliferation of myofibroblasts and adipocytes, and excessive secretion of hyaluronic acid. Targeted therapeutic strategies based on this intricate pathophysiological model would naturally involve interventions aimed at preventing T cell activation and depletion^43^.

Agents targeting CD3, including ciclosporin, otelixizumab, and teplizumab, have demonstrated significant potential in attenuating T cell activity. Additionally, rituximab treatment offers an adjunctive therapeutic approach for B cell depletion^44^. Notably, the inhibition of cytokines, exemplified by tocilizumab, and the utilization of anti-TNF alpha monoclonal antibodies have been investigated^45^. Moreover, the comprehensive suppression of immune cell proliferation through the use of antimetabolites such as azathioprine and mycophenolate has shown promising outcomes in studies of TED therapy^46^. Expanding upon previous investigations, we further explored the expression of PMGs within the immune microenvironment. T cells, follicular helper cells, and neutrophils were found to exhibit upregulated expression in the treatment group. These findings provide additional evidence for the involvement of PMGs in the pathogenesis of TED, particularly with respect to inflammation and immune response.

The quest to identify biomarkers and their interrelationships with TED remains an understudied domain in extant scientific literature. Contemporary research endeavors, employing bioinformatic analyses, have begun to elucidate the metabolic correlates of ocular maladies^47–49^. For instance, studies by Liu et al. deployed Weighted Gene Coexpression Network Analysis to pinpoint hub genes integral to TED. Concurrently, Hu et al. innovatively fashioned a bioinformatic model to identify a cadre of 11 salient genes implicated in thyroid eye disease, including ATP6V1A and PTGES3 among others. In a related vein, Huang et al. discerned six significant genes for diabetic retinopathy through an amalgamation of comprehensive bioinformatics scrutiny and in vivo validation, including CD44 and CDC42, to name a few. Remarkably, the nexus between Purine Metabolism and TED remains a terra incognita in the research landscape.

In the burgeoning frontier of cancer immunotherapy, this investigation meticulously delineates a cardinal function for PMGs, engendering empirical correlations with pivotal immunological indicators and thereby, enriching our apprehension of its immunomodulatory interactions. The findings not only amplify the prevailing paradigm of PMGs' involvement in the intricate interplay between TED and host immunity but also carve out a novel scientific trajectory, intertwining detailed molecular insights with clinical relevance. Furthermore, this work forges ahead, providing clinicians with invaluable insights, potentially sculpting the future framework for a more nuanced understanding and therapeutic strategy toward TED, thereby fostering an enriched clinical insight that is anticipated to steer future exploratory and therapeutic endeavours in the entwined realms of immunotherapy and ocular pathophysiology. However, it is critical to recognize the constraints on our study. First, the integrity of our verified PMGs prognostic signature is inextricably linked to GEO datasets, emphasizing the importance of independent validation via external data repositories—resources that have proven difficult in the current context. Second, while our bioinformatics analysis provides important early insights into the functional dynamics of PMGs in oncological contexts, these computational discoveries are simply the starting point for a more detailed investigation. There is still an urgent need for empirical confirmation via rigorous in vitro and in vivo experimental designs to transform these preliminary findings into effective treatment solutions. Finally, the post-translational landscape—important in altering intracellular signaling cascades and regulatory molecule functional activities—represents a gap in our current understanding of PMGs. Current databases provide insufficient information on these crucial biochemical alterations, indicating a need for more research.

## Conclusions

The etiology and pathogenesis of TED are orchestrated by a complex interplay of numerous molecular targets, cellular pathways, signal transduction mechanisms, and regulatory networks, which manifest as both synergistic and bidirectional modulatory effects. Within the cohort of PMGs examined, pivotal regulators, notably PRPS2, PFAS, ATIC, NT5C1A, POLR2E, POLR2F, POLR3B, PDE3A, ADSS, and NTPCR, have been delineated. Singular emphasis has been placed on PFAS and POLR2F for their salient roles in disease dynamics. This investigation augments our nuanced understanding of the intricate interrelations between PMGs and TED, thereby charting a course for future innovations in diagnostic modalities and therapeutic interventions for this multifaceted disorder.

## Data availability

The datasets generated during and/or analyzed during the current study are available in the appendix.

### Supplementary Information

Supplementary Information.

## Abbreviations

TED: Thyroid eye disease
GO: Gene ontology
TCM: Traditional Chinese medicine
MF: Molecular functions
KEGG: Kyoto encyclopedia of genes and genomes
GEO: Gene expression omnibus
PMGs: Purine metabolism genes
BP: Biological processes
CC: Cellular components
DEGs: Differentially expressed genes
