# Supplementary material for: Purine metabolism-related genes and immunization in thyroid eye disease were validated using bioinformatics and machine learning
Source: Sci Rep. 2023 Oct 26;13:18391. doi: 10.1038/s41598-023-45048-9 (PMC10603126; doi:10.1038/s41598-023-45048-9)
Supplement: Supplementary file 1 — Supplementary Information. [file 41598_2023_45048_MOESM1_ESM.doc]

Purine Metabolism-Related Genes and Immunization in Thyroid Eye Disease were Validated using Bioinformatics and Machine Learning

**Supplementary appendix to the manuscript**

Contents of supplementary appendix

[Appendix 1 3](#__RefHeading___Toc26846)

[Datasets and Purine Metabolism genes 3](#__RefHeading___Toc22445)

[Table S1. Purine Metabolism genes 3](#__RefHeading___Toc22909)

[Appendix 2 5](#__RefHeading___Toc12868)

[DEGs linked to Purine Metabolism genes 5](#__RefHeading___Toc29305)

[Table S2. 65 DEGs linked to Purine Metabolism genes. 5](#__RefHeading___Toc25263)

[Appendix 3 8](#__RefHeading___Toc3647)

[Table S3a. Analysis of GO. 8](#__RefHeading___Toc10978)

[Table S3b. Analysis of KEGG. 39](#__RefHeading___Toc31537)

[Appendix 4 41](#__RefHeading___Toc19218)

[Table S4a. LASSO genes. 41](#__RefHeading___Toc16052)

[Table S4b. SVM-RFE genes. 41](#__RefHeading___Toc2282)

[Table S4c. InterGenes. 41](#__RefHeading___Toc3622)

[Appendix 5 42](#__RefHeading___Toc6470)

[Table 5a. PFAS of GSEA analysis. 42](#__RefHeading___Toc5148)

[Table 5b. POLR2F of GSEA analysis. 47](#__RefHeading___Toc18664)

[Appendix 6 53](#__RefHeading___Toc3274)

[Table 6. Drug prediction. 53](#__RefHeading___Toc25544)

[Appendix 7 55](#__RefHeading___Toc23181)

[Table 7a. Gene-miRNA. 55](#__RefHeading___Toc6126)

[Table 7b. Gene-lncRNA. 62](#__RefHeading___Toc31728)

# Appendix 1

**Datasets and Purine Metabolism genes**

**Table S1. Purine Metabolism genes**

| NUDT9 | POLA1 | ZNRD1 | AK2 | ENTPD4 | PDE6B |
| --- | --- | --- | --- | --- | --- |
| ADPRM | POLA2 | TWISTNB | AK1 | ENTPD5 | PDE6C |
| NUDT5 | PRIM1 | POLR1E | PDE6A | ENTPD6 | PDE6D |
| PGM1 | PRIM2 | POLR2A | AK8 | NUDT16 | PDE6G |
| PGM2 | POLD1 | POLR2B | AK6 | ITPA | PDE6H |
| PRPS1L1 | POLD2 | POLR2C | AK3 | XDH | PDE9A |
| PRPS2 | POLD3 | POLR2D | ENTPD2 | NUDT2 | PDE10A |
| PRPS1 | POLD4 | POLR2E | NTPCR | GMPS | PDE11A |
| PPAT | POLE | POLR2F | PNPT1 | GMPR | ADSSL1 |
| GART | POLE2 | POLR2G | PDE4A | GMPR2 | ADSS |
| PFAS | POLE3 | POLR2H | PDE4B | GDA | AMPD2 |
| PAICS | POLE4 | POLR2I | PDE4C | GUK1 | AMPD3 |
| ADSL | HDDC3 | POLR2L | PDE4D | PKM | AMPD1 |
| ATIC | PRUNE1 | POLR2J | PDE7A | PKLR | ADK |
| APRT | ADCY1 | POLR2J3 | PDE7B | RRM1 | DCK |
| NT5C2 | ADCY2 | POLR2J2 | PDE8B | RRM2B | ADA |
| NT5C1A | ADCY3 | POLR2K | PDE8A | RRM2 | CECR1 |
| NT5C1B | ADCY4 | POLR3A | FHIT | DGUOK | AK7 |
| NT5C | ADCY5 | POLR3B | ENPP4 | POLR1A | AK4 |
| NT5M | ADCY6 | POLR3C | PAPSS2 | POLR1B | AK5 |
| NT5C3A | ADCY7 | POLR3D | PAPSS1 | NME2 | NPR1 |
| NT5C3B | ADCY8 | POLR3E | ENPP1 | NME4 | NPR2 |
| NT5C1B-RDH14 | ADCY9 | RPC5 | ENPP3 | NME1 | PDE1A |
| NT5E | ADCY10 | POLR1C | URAD | NME3 | PDE1B |
| PNP | GUCY1A2 | POLR3K | ALLC | NME1-NME2 | PDE1C |
| HPRT1 | GUCY1A3 | POLR1D | NME7 | AK9 | PDE2A |
| IMPDH1 | GUCY1B3 | POLR3H | GUCY2F | ENTPD3 | PDE3A |
| IMPDH2 | GUCY2C | POLR3GL | POLR3F | ENTPD8 | PDE3B |
| NME6 | GUCY2D | POLR3G | CANT1 | ENTPD1 | PDE5A |

# Appendix 2

## **DEGs linked to Purine Metabolism genes**

**Table S2. 65 DEGs linked to Purine Metabolism genes.**

| Gene | conMean | treatMean | pvalue | Type |
| --- | --- | --- | --- | --- |
| NUDT5 | 4.63083969 | 4.389776343 | 0.001682262 | Down |
| PRPS1L1 | 1.928407793 | 2.082882 | 0.042382031 | Up |
| PRPS2 | 3.035256552 | 2.655193571 | 0.000192883 | Down |
| PRPS1 | 5.584498345 | 5.298372371 | 0.002463692 | Down |
| PPAT | 2.838694379 | 2.6693988 | 0.012870907 | Down |
| PFAS | 4.444799793 | 4.176914371 | 0.045405918 | Down |
| ADSL | 5.557542552 | 5.288342086 | 0.002597436 | Down |
| ATIC | 6.717000172 | 6.3897062 | 0.00118456 | Down |
| APRT | 6.274149586 | 6.471775943 | 0.039712909 | Up |
| NT5C2 | 6.851611069 | 6.384775914 | 0.002722795 | Down |
| NT5C1A | 3.040212517 | 3.529918314 | 1.72E-06 | Up |
| NT5C | 5.853547276 | 6.110757686 | 0.017529876 | Up |
| NT5M | 4.21912231 | 4.507759029 | 0.040366448 | Up |
| HPRT1 | 2.779909241 | 2.553073629 | 0.035977396 | Down |
| IMPDH2 | 7.776686552 | 7.484381486 | 0.000644466 | Down |
| NME1 | 5.488439828 | 5.210044457 | 0.024446751 | Down |
| NME3 | 6.067005034 | 6.433969114 | 0.015635417 | Up |
| AK9 | 3.841352172 | 3.613627686 | 0.009719614 | Down |
| ENTPD4 | 5.401786552 | 5.109872914 | 0.024446751 | Down |
| GMPS | 4.609444448 | 4.3708182 | 0.02921147 | Down |
| GUK1 | 6.567779448 | 6.787964343 | 0.013080049 | Up |
| PKLR | 3.172165241 | 3.416504657 | 0.001015628 | Up |
| DGUOK | 4.911737862 | 4.7477168 | 0.021128266 | Down |
| POLR1B | 3.596367517 | 3.3806392 | 0.019992775 | Down |
| ZNRD1 | 3.887505931 | 4.090355943 | 0.014473323 | Up |
| TWISTNB | 2.485807414 | 2.2975746 | 0.027218708 | Down |
| POLR1E | 4.654428862 | 4.850176057 | 0.006125989 | Up |
| POLR2B | 5.237632483 | 4.9275014 | 0.021919386 | Down |
| POLR2C | 6.127909379 | 5.8865254 | 0.028200154 | Down |
| POLR2E | 7.822311724 | 7.623402057 | 0.023041603 | Down |
| POLR2F | 5.975729759 | 6.130828829 | 0.035946329 | Up |
| POLR2G | 6.075263 | 5.824037343 | 0.017293684 | Down |
| POLR2I | 8.273061586 | 7.932369143 | 0.021096491 | Down |
| POLR2J2 | 6.943610931 | 7.3913704 | 0.006395199 | Up |
| POLR2K | 5.719249586 | 5.276701429 | 0.003281043 | Down |
| POLR3B | 2.656412517 | 2.4801858 | 0.006395199 | Down |
| POLR3C | 4.522248897 | 4.316211971 | 0.02921147 | Down |
| POLR3E | 3.322319 | 3.537316971 | 0.019619278 | Up |
| POLR1C | 4.578863517 | 4.867073029 | 0.018900194 | Up |
| POLR1D | 5.384956345 | 4.960856486 | 0.033569524 | Down |
| POLA1 | 4.548826207 | 4.144775571 | 0.007277877 | Down |
| PRIM1 | 3.191603621 | 2.721859971 | 5.23E-05 | Down |
| POLE | 5.818999069 | 6.121637257 | 0.012870907 | Up |
| POLE3 | 3.163984414 | 2.643286771 | 0.001378649 | Down |
| ADCY1 | 3.751718276 | 3.956916743 | 0.019619278 | Up |
| ADCY8 | 4.309646103 | 4.838597257 | 0.025342682 | Up |
| ADCY10 | 3.058459 | 3.272607029 | 0.030253354 | Up |
| PDE3A | 3.947643448 | 4.207756743 | 0.005377635 | Up |
| PDE6A | 4.435619345 | 4.679809771 | 0.016877485 | Up |
| PDE6D | 4.408674759 | 4.217238057 | 0.008241585 | Down |
| PDE6G | 5.252006138 | 5.483534943 | 0.017611451 | Up |
| ADSSL1 | 4.352285862 | 4.506761914 | 0.0425131 | Up |
| ADSS | 3.891887276 | 3.657009543 | 0.014918543 | Down |
| ADK | 3.991337966 | 3.608342086 | 0.001714683 | Down |
| DCK | 2.909242276 | 2.695049171 | 0.027446959 | Down |
| CECR1 | 6.584831517 | 5.981532886 | 0.006965234 | Down |
| AK5 | 4.146779241 | 4.385131286 | 0.037186761 | Up |
| AK2 | 5.438494379 | 5.228843657 | 0.048460292 | Down |
| AK6 | 5.49614031 | 4.991983571 | 0.007266748 | Down |
| AK3 | 5.28362931 | 4.736800914 | 0.006395199 | Down |
| ENTPD2 | 5.053020276 | 5.4637546 | 0.001023402 | Up |
| NTPCR | 4.748656966 | 4.393701257 | 0.000192883 | Down |
| ENPP4 | 2.400151172 | 2.166763771 | 0.048460292 | Down |
| PAPSS1 | 6.111313862 | 5.588505714 | 0.020361768 | Down |
| ENPP1 | 3.562861414 | 3.219453486 | 0.001922554 | Down |

# Appendix 3

**Analysis of enrichment**

**Table S3a. Analysis of GO.**

| ONTOLOGY | ID | Description | BgRatio | pvalue | qvalue | Count |
| --- | --- | --- | --- | --- | --- | --- |
| BP | GO:0009123 | nucleoside monophosphate metabolic process | 75/18862 | 1.87E-41 | 9.02E-39 | 23 |
| BP | GO:0009126 | purine nucleoside monophosphate metabolic process | 43/18862 | 7.59E-38 | 1.83E-35 | 19 |
| BP | GO:0006164 | purine nucleotide biosynthetic process | 197/18862 | 1.66E-36 | 2.66E-34 | 26 |
| BP | GO:0072521 | purine-containing compound metabolic process | 460/18862 | 2.21E-36 | 2.66E-34 | 32 |
| BP | GO:0072522 | purine-containing compound biosynthetic process | 208/18862 | 7.37E-36 | 7.09E-34 | 26 |
| BP | GO:0009116 | nucleoside metabolic process | 104/18862 | 1.46E-35 | 1.17E-33 | 22 |
| BP | GO:0006163 | purine nucleotide metabolic process | 441/18862 | 2.88E-35 | 1.98E-33 | 31 |
| BP | GO:0009165 | nucleotide biosynthetic process | 264/18862 | 7.38E-35 | 4.44E-33 | 27 |
| BP | GO:1901293 | nucleoside phosphate biosynthetic process | 267/18862 | 1.01E-34 | 5.41E-33 | 27 |
| BP | GO:0019693 | ribose phosphate metabolic process | 435/18862 | 9.11E-34 | 4.38E-32 | 30 |
| BP | GO:1901657 | glycosyl compound metabolic process | 129/18862 | 2.60E-33 | 1.14E-31 | 22 |
| BP | GO:0046390 | ribose phosphate biosynthetic process | 195/18862 | 8.20E-33 | 3.29E-31 | 24 |
| BP | GO:0009161 | ribonucleoside monophosphate metabolic process | 56/18862 | 8.98E-33 | 3.32E-31 | 18 |
| BP | GO:0009167 | purine ribonucleoside monophosphate metabolic process | 40/18862 | 5.37E-31 | 1.85E-29 | 16 |
| BP | GO:0009152 | purine ribonucleotide biosynthetic process | 175/18862 | 3.30E-30 | 1.06E-28 | 22 |
| BP | GO:0009150 | purine ribonucleotide metabolic process | 408/18862 | 1.21E-29 | 3.65E-28 | 27 |
| BP | GO:0009260 | ribonucleotide biosynthetic process | 188/18862 | 1.72E-29 | 4.86E-28 | 22 |
| BP | GO:0009259 | ribonucleotide metabolic process | 425/18862 | 3.68E-29 | 9.84E-28 | 27 |
| BP | GO:0009124 | nucleoside monophosphate biosynthetic process | 43/18862 | 1.19E-25 | 3.00E-24 | 14 |
| BP | GO:0009127 | purine nucleoside monophosphate biosynthetic process | 23/18862 | 3.50E-25 | 8.41E-24 | 12 |
| BP | GO:0046128 | purine ribonucleoside metabolic process | 58/18862 | 1.48E-23 | 3.40E-22 | 14 |
| BP | GO:0042278 | purine nucleoside metabolic process | 61/18862 | 3.27E-23 | 7.16E-22 | 14 |
| BP | GO:0034404 | nucleobase-containing small molecule biosynthetic process | 115/18862 | 1.09E-22 | 2.28E-21 | 16 |
| BP | GO:0009156 | ribonucleoside monophosphate biosynthetic process | 34/18862 | 1.38E-22 | 2.77E-21 | 12 |
| BP | GO:0009119 | ribonucleoside metabolic process | 72/18862 | 4.24E-22 | 8.16E-21 | 14 |
| BP | GO:0009168 | purine ribonucleoside monophosphate biosynthetic process | 21/18862 | 1.38E-20 | 2.55E-19 | 10 |
| BP | GO:1901068 | guanosine-containing compound metabolic process | 40/18862 | 3.84E-15 | 6.84E-14 | 9 |
| BP | GO:0009151 | purine deoxyribonucleotide metabolic process | 14/18862 | 6.84E-15 | 1.13E-13 | 7 |
| BP | GO:0046040 | IMP metabolic process | 14/18862 | 6.84E-15 | 1.13E-13 | 7 |
| BP | GO:0046033 | AMP metabolic process | 16/18862 | 2.27E-14 | 3.64E-13 | 7 |
| BP | GO:0009141 | nucleoside triphosphate metabolic process | 109/18862 | 3.18E-14 | 4.93E-13 | 11 |
| BP | GO:0006220 | pyrimidine nucleotide metabolic process | 50/18862 | 3.43E-14 | 5.16E-13 | 9 |
| BP | GO:0072527 | pyrimidine-containing compound metabolic process | 85/18862 | 1.05E-13 | 1.53E-12 | 10 |
| BP | GO:0009394 | 2'-deoxyribonucleotide metabolic process | 36/18862 | 1.58E-13 | 2.24E-12 | 8 |
| BP | GO:0009163 | nucleoside biosynthetic process | 38/18862 | 2.54E-13 | 3.40E-12 | 8 |
| BP | GO:0019692 | deoxyribose phosphate metabolic process | 38/18862 | 2.54E-13 | 3.40E-12 | 8 |
| BP | GO:0042451 | purine nucleoside biosynthetic process | 22/18862 | 3.33E-13 | 4.11E-12 | 7 |
| BP | GO:0042455 | ribonucleoside biosynthetic process | 22/18862 | 3.33E-13 | 4.11E-12 | 7 |
| BP | GO:0046129 | purine ribonucleoside biosynthetic process | 22/18862 | 3.33E-13 | 4.11E-12 | 7 |
| BP | GO:0009262 | deoxyribonucleotide metabolic process | 40/18862 | 3.98E-13 | 4.79E-12 | 8 |
| BP | GO:1901659 | glycosyl compound biosynthetic process | 42/18862 | 6.08E-13 | 7.13E-12 | 8 |
| BP | GO:0050434 | positive regulation of viral transcription | 26/18862 | 1.27E-12 | 1.46E-11 | 7 |
| BP | GO:0015949 | nucleobase-containing small molecule interconversion | 27/18862 | 1.71E-12 | 1.89E-11 | 7 |
| BP | GO:0009132 | nucleoside diphosphate metabolic process | 156/18862 | 1.73E-12 | 1.89E-11 | 11 |
| BP | GO:0034656 | nucleobase-containing small molecule catabolic process | 50/18862 | 2.71E-12 | 2.90E-11 | 8 |
| BP | GO:0006354 | DNA-templated transcription, elongation | 119/18862 | 3.29E-12 | 3.45E-11 | 10 |
| BP | GO:1901292 | nucleoside phosphate catabolic process | 83/18862 | 4.16E-12 | 4.26E-11 | 9 |
| BP | GO:0009142 | nucleoside triphosphate biosynthetic process | 84/18862 | 4.65E-12 | 4.66E-11 | 9 |
| BP | GO:0006370 | 7-methylguanosine mRNA capping | 33/18862 | 8.13E-12 | 7.98E-11 | 7 |
| BP | GO:0009452 | 7-methylguanosine RNA capping | 34/18862 | 1.02E-11 | 9.63E-11 | 7 |
| BP | GO:0036260 | RNA capping | 34/18862 | 1.02E-11 | 9.63E-11 | 7 |
| BP | GO:0046782 | regulation of viral transcription | 42/18862 | 5.02E-11 | 4.61E-10 | 7 |
| BP | GO:0098781 | ncRNA transcription | 109/18862 | 5.07E-11 | 4.61E-10 | 9 |
| BP | GO:0006188 | IMP biosynthetic process | 10/18862 | 6.27E-11 | 5.48E-10 | 5 |
| BP | GO:0009113 | purine nucleobase biosynthetic process | 10/18862 | 6.27E-11 | 5.48E-10 | 5 |
| BP | GO:0009265 | 2'-deoxyribonucleotide biosynthetic process | 12/18862 | 1.96E-10 | 1.65E-09 | 5 |
| BP | GO:0046385 | deoxyribose phosphate biosynthetic process | 12/18862 | 1.96E-10 | 1.65E-09 | 5 |
| BP | GO:0009144 | purine nucleoside triphosphate metabolic process | 86/18862 | 2.46E-10 | 2.04E-09 | 8 |
| BP | GO:0009199 | ribonucleoside triphosphate metabolic process | 87/18862 | 2.70E-10 | 2.20E-09 | 8 |
| BP | GO:0046940 | nucleoside monophosphate phosphorylation | 13/18862 | 3.18E-10 | 2.51E-09 | 5 |
| BP | GO:1901070 | guanosine-containing compound biosynthetic process | 13/18862 | 3.18E-10 | 2.51E-09 | 5 |
| BP | GO:0009218 | pyrimidine ribonucleotide metabolic process | 29/18862 | 3.24E-10 | 2.51E-09 | 6 |
| BP | GO:0006362 | transcription elongation from RNA polymerase I promoter | 30/18862 | 4.04E-10 | 3.08E-09 | 6 |
| BP | GO:0009185 | ribonucleoside diphosphate metabolic process | 138/18862 | 4.25E-10 | 3.20E-09 | 9 |
| BP | GO:0006363 | termination of RNA polymerase I transcription | 31/18862 | 5.00E-10 | 3.70E-09 | 6 |
| BP | GO:0009263 | deoxyribonucleotide biosynthetic process | 15/18862 | 7.38E-10 | 5.30E-09 | 5 |
| BP | GO:0043101 | purine-containing compound salvage | 15/18862 | 7.38E-10 | 5.30E-09 | 5 |
| BP | GO:0009164 | nucleoside catabolic process | 34/18862 | 9.06E-10 | 6.41E-09 | 6 |
| BP | GO:0046434 | organophosphate catabolic process | 154/18862 | 1.13E-09 | 7.88E-09 | 9 |
| BP | GO:0006289 | nucleotide-excision repair | 108/18862 | 1.55E-09 | 1.06E-08 | 8 |
| BP | GO:0009145 | purine nucleoside triphosphate biosynthetic process | 68/18862 | 1.70E-09 | 1.15E-08 | 7 |
| BP | GO:0006361 | transcription initiation from RNA polymerase I promoter | 38/18862 | 1.84E-09 | 1.23E-08 | 6 |
| BP | GO:0046112 | nucleobase biosynthetic process | 18/18862 | 2.09E-09 | 1.38E-08 | 5 |
| BP | GO:0035019 | somatic stem cell population maintenance | 71/18862 | 2.31E-09 | 1.50E-08 | 7 |
| BP | GO:0006283 | transcription-coupled nucleotide-excision repair | 73/18862 | 2.82E-09 | 1.79E-08 | 7 |
| BP | GO:0006144 | purine nucleobase metabolic process | 19/18862 | 2.83E-09 | 1.79E-08 | 5 |
| BP | GO:0042795 | snRNA transcription by RNA polymerase II | 74/18862 | 3.10E-09 | 1.94E-08 | 7 |
| BP | GO:0009301 | snRNA transcription | 75/18862 | 3.41E-09 | 2.10E-08 | 7 |
| BP | GO:0032481 | positive regulation of type I interferon production | 77/18862 | 4.11E-09 | 2.51E-08 | 7 |
| BP | GO:1901658 | glycosyl compound catabolic process | 44/18862 | 4.64E-09 | 2.79E-08 | 6 |
| BP | GO:0006352 | DNA-templated transcription, initiation | 249/18862 | 4.78E-09 | 2.84E-08 | 10 |
| BP | GO:0009205 | purine ribonucleoside triphosphate metabolic process | 80/18862 | 5.40E-09 | 3.17E-08 | 7 |
| BP | GO:0032479 | regulation of type I interferon production | 128/18862 | 5.98E-09 | 3.47E-08 | 8 |
| BP | GO:0032606 | type I interferon production | 129/18862 | 6.36E-09 | 3.64E-08 | 8 |
| BP | GO:0009135 | purine nucleoside diphosphate metabolic process | 135/18862 | 9.12E-09 | 5.10E-08 | 8 |
| BP | GO:0009179 | purine ribonucleoside diphosphate metabolic process | 135/18862 | 9.12E-09 | 5.10E-08 | 8 |
| BP | GO:0072523 | purine-containing compound catabolic process | 51/18862 | 1.16E-08 | 6.44E-08 | 6 |
| BP | GO:0006368 | transcription elongation from RNA polymerase II promoter | 90/18862 | 1.24E-08 | 6.77E-08 | 7 |
| BP | GO:0048524 | positive regulation of viral process | 91/18862 | 1.34E-08 | 7.24E-08 | 7 |
| BP | GO:0009133 | nucleoside diphosphate biosynthetic process | 10/18862 | 1.79E-08 | 9.55E-08 | 4 |
| BP | GO:0045815 | positive regulation of gene expression, epigenetic | 58/18862 | 2.57E-08 | 1.36E-07 | 6 |
| BP | GO:0008543 | fibroblast growth factor receptor signaling pathway | 113/18862 | 6.06E-08 | 3.17E-07 | 7 |
| BP | GO:0009206 | purine ribonucleoside triphosphate biosynthetic process | 67/18862 | 6.21E-08 | 3.21E-07 | 6 |
| BP | GO:0009112 | nucleobase metabolic process | 34/18862 | 6.54E-08 | 3.31E-07 | 5 |
| BP | GO:0043094 | cellular metabolic compound salvage | 34/18862 | 6.54E-08 | 3.31E-07 | 5 |
| BP | GO:0006360 | transcription by RNA polymerase I | 68/18862 | 6.80E-08 | 3.41E-07 | 6 |
| BP | GO:0060964 | regulation of gene silencing by miRNA | 121/18862 | 9.71E-08 | 4.82E-07 | 7 |
| BP | GO:0009166 | nucleotide catabolic process | 73/18862 | 1.04E-07 | 5.07E-07 | 6 |
| BP | GO:0009201 | ribonucleoside triphosphate biosynthetic process | 73/18862 | 1.04E-07 | 5.07E-07 | 6 |
| BP | GO:0046037 | GMP metabolic process | 15/18862 | 1.15E-07 | 5.48E-07 | 4 |
| BP | GO:0060147 | regulation of posttranscriptional gene silencing | 124/18862 | 1.15E-07 | 5.48E-07 | 7 |
| BP | GO:0060966 | regulation of gene silencing by RNA | 125/18862 | 1.21E-07 | 5.73E-07 | 7 |
| BP | GO:0006353 | DNA-templated transcription, termination | 75/18862 | 1.23E-07 | 5.74E-07 | 6 |
| BP | GO:0072528 | pyrimidine-containing compound biosynthetic process | 40/18862 | 1.52E-07 | 7.00E-07 | 5 |
| BP | GO:0043174 | nucleoside salvage | 16/18862 | 1.53E-07 | 7.00E-07 | 4 |
| BP | GO:0009208 | pyrimidine ribonucleoside triphosphate metabolic process | 18/18862 | 2.56E-07 | 1.16E-06 | 4 |
| BP | GO:0044344 | cellular response to fibroblast growth factor stimulus | 142/18862 | 2.91E-07 | 1.31E-06 | 7 |
| BP | GO:0006195 | purine nucleotide catabolic process | 46/18862 | 3.13E-07 | 1.39E-06 | 5 |
| BP | GO:0060968 | regulation of gene silencing | 144/18862 | 3.20E-07 | 1.41E-06 | 7 |
| BP | GO:0019827 | stem cell population maintenance | 146/18862 | 3.51E-07 | 1.54E-06 | 7 |
| BP | GO:0071774 | response to fibroblast growth factor | 148/18862 | 3.85E-07 | 1.65E-06 | 7 |
| BP | GO:0098727 | maintenance of cell number | 148/18862 | 3.85E-07 | 1.65E-06 | 7 |
| BP | GO:0044282 | small molecule catabolic process | 431/18862 | 8.08E-07 | 3.44E-06 | 10 |
| BP | GO:0046039 | GTP metabolic process | 24/18862 | 8.75E-07 | 3.69E-06 | 4 |
| BP | GO:0046148 | pigment biosynthetic process | 57/18862 | 9.31E-07 | 3.89E-06 | 5 |
| BP | GO:0009147 | pyrimidine nucleoside triphosphate metabolic process | 25/18862 | 1.04E-06 | 4.31E-06 | 4 |
| BP | GO:0032201 | telomere maintenance via semi-conservative replication | 27/18862 | 1.44E-06 | 5.87E-06 | 4 |
| BP | GO:0019083 | viral transcription | 180/18862 | 1.44E-06 | 5.87E-06 | 7 |
| BP | GO:0050792 | regulation of viral process | 186/18862 | 1.79E-06 | 7.24E-06 | 7 |
| BP | GO:0006367 | transcription initiation from RNA polymerase II promoter | 187/18862 | 1.86E-06 | 7.45E-06 | 7 |
| BP | GO:0006221 | pyrimidine nucleotide biosynthetic process | 30/18862 | 2.23E-06 | 8.85E-06 | 4 |
| BP | GO:0043903 | regulation of biological process involved in symbiotic interaction | 197/18862 | 2.63E-06 | 1.04E-05 | 7 |
| BP | GO:0019080 | viral gene expression | 198/18862 | 2.72E-06 | 1.06E-05 | 7 |
| BP | GO:0042440 | pigment metabolic process | 72/18862 | 3.00E-06 | 1.16E-05 | 5 |
| BP | GO:0006152 | purine nucleoside catabolic process | 10/18862 | 3.43E-06 | 1.30E-05 | 3 |
| BP | GO:0006171 | cAMP biosynthetic process | 10/18862 | 3.43E-06 | 1.30E-05 | 3 |
| BP | GO:0046130 | purine ribonucleoside catabolic process | 10/18862 | 3.43E-06 | 1.30E-05 | 3 |
| BP | GO:0006183 | GTP biosynthetic process | 11/18862 | 4.71E-06 | 1.76E-05 | 3 |
| BP | GO:0006213 | pyrimidine nucleoside metabolic process | 36/18862 | 4.72E-06 | 1.76E-05 | 4 |
| BP | GO:0009134 | nucleoside diphosphate catabolic process | 12/18862 | 6.27E-06 | 2.30E-05 | 3 |
| BP | GO:0046051 | UTP metabolic process | 12/18862 | 6.27E-06 | 2.30E-05 | 3 |
| BP | GO:0006270 | DNA replication initiation | 40/18862 | 7.25E-06 | 2.62E-05 | 4 |
| BP | GO:0072529 | pyrimidine-containing compound catabolic process | 40/18862 | 7.25E-06 | 2.62E-05 | 4 |
| BP | GO:0009125 | nucleoside monophosphate catabolic process | 13/18862 | 8.13E-06 | 2.92E-05 | 3 |
| BP | GO:0043173 | nucleotide salvage | 14/18862 | 1.03E-05 | 3.68E-05 | 3 |
| BP | GO:0009200 | deoxyribonucleoside triphosphate metabolic process | 16/18862 | 1.58E-05 | 5.55E-05 | 3 |
| BP | GO:0046036 | CTP metabolic process | 16/18862 | 1.58E-05 | 5.55E-05 | 3 |
| BP | GO:0009162 | deoxyribonucleoside monophosphate metabolic process | 17/18862 | 1.92E-05 | 6.68E-05 | 3 |
| BP | GO:0006271 | DNA strand elongation involved in DNA replication | 19/18862 | 2.72E-05 | 9.41E-05 | 3 |
| BP | GO:0006244 | pyrimidine nucleotide catabolic process | 20/18862 | 3.19E-05 | 0.000109644 | 3 |
| BP | GO:0033260 | nuclear DNA replication | 59/18862 | 3.46E-05 | 0.000117902 | 4 |
| BP | GO:1901136 | carbohydrate derivative catabolic process | 198/18862 | 3.52E-05 | 0.000119193 | 6 |
| BP | GO:0046031 | ADP metabolic process | 122/18862 | 3.95E-05 | 0.0001328 | 5 |
| BP | GO:0040029 | regulation of gene expression, epigenetic | 205/18862 | 4.27E-05 | 0.000139487 | 6 |
| BP | GO:0009190 | cyclic nucleotide biosynthetic process | 22/18862 | 4.29E-05 | 0.000139487 | 3 |
| BP | GO:0009220 | pyrimidine ribonucleotide biosynthetic process | 22/18862 | 4.29E-05 | 0.000139487 | 3 |
| BP | GO:0046058 | cAMP metabolic process | 22/18862 | 4.29E-05 | 0.000139487 | 3 |
| BP | GO:0052652 | cyclic purine nucleotide metabolic process | 22/18862 | 4.29E-05 | 0.000139487 | 3 |
| BP | GO:0044786 | cell cycle DNA replication | 64/18862 | 4.77E-05 | 0.000153956 | 4 |
| BP | GO:0006541 | glutamine metabolic process | 23/18862 | 4.92E-05 | 0.000156873 | 3 |
| BP | GO:0046135 | pyrimidine nucleoside catabolic process | 23/18862 | 4.92E-05 | 0.000156873 | 3 |
| BP | GO:0046034 | ATP metabolic process | 313/18862 | 5.28E-05 | 0.000167007 | 7 |
| BP | GO:0042454 | ribonucleoside catabolic process | 24/18862 | 5.62E-05 | 0.000176547 | 3 |
| BP | GO:0006165 | nucleoside diphosphate phosphorylation | 132/18862 | 5.75E-05 | 0.000179681 | 5 |
| BP | GO:0046939 | nucleotide phosphorylation | 133/18862 | 5.96E-05 | 0.000185054 | 5 |
| BP | GO:0022616 | DNA strand elongation | 26/18862 | 7.18E-05 | 0.000221441 | 3 |
| BP | GO:0009154 | purine ribonucleotide catabolic process | 35/18862 | 0.000177189 | 0.000542913 | 3 |
| BP | GO:0000377 | RNA splicing, via transesterification reactions with bulged adenosine as nucleophile | 383/18862 | 0.000185263 | 0.000560512 | 7 |
| BP | GO:0000398 | mRNA splicing, via spliceosome | 383/18862 | 0.000185263 | 0.000560512 | 7 |
| BP | GO:0000375 | RNA splicing, via transesterification reactions | 386/18862 | 0.000194332 | 0.000584273 | 7 |
| BP | GO:0009187 | cyclic nucleotide metabolic process | 40/18862 | 0.000264521 | 0.000785486 | 3 |
| BP | GO:0009261 | ribonucleotide catabolic process | 40/18862 | 0.000264521 | 0.000785486 | 3 |
| BP | GO:0007595 | lactation | 46/18862 | 0.000401043 | 0.001183576 | 3 |
| BP | GO:0001819 | positive regulation of cytokine production | 437/18862 | 0.000412087 | 0.001208754 | 7 |
| BP | GO:0006228 | UTP biosynthetic process | 10/18862 | 0.00042593 | 0.001223928 | 2 |
| BP | GO:1904321 | response to forskolin | 10/18862 | 0.00042593 | 0.001223928 | 2 |
| BP | GO:1904322 | cellular response to forskolin | 10/18862 | 0.00042593 | 0.001223928 | 2 |
| BP | GO:0006383 | transcription by RNA polymerase III | 47/18862 | 0.000427437 | 0.001223928 | 3 |
| BP | GO:0046710 | GDP metabolic process | 11/18862 | 0.000519534 | 0.001478032 | 2 |
| BP | GO:0071375 | cellular response to peptide hormone stimulus | 325/18862 | 0.000522324 | 0.001478032 | 6 |
| BP | GO:0006754 | ATP biosynthetic process | 56/18862 | 0.000716275 | 0.002015006 | 3 |
| BP | GO:0008380 | RNA splicing | 481/18862 | 0.000728322 | 0.002036983 | 7 |
| BP | GO:0006241 | CTP biosynthetic process | 14/18862 | 0.000854418 | 0.002375838 | 2 |
| BP | GO:0009209 | pyrimidine ribonucleoside triphosphate biosynthetic process | 15/18862 | 0.000983885 | 0.002720118 | 2 |
| BP | GO:0007625 | grooming behavior | 16/18862 | 0.001122181 | 0.003084732 | 2 |
| BP | GO:1901653 | cellular response to peptide | 391/18862 | 0.001362125 | 0.003723034 | 6 |
| BP | GO:0009223 | pyrimidine deoxyribonucleotide catabolic process | 18/18862 | 0.001425039 | 0.003851229 | 2 |
| BP | GO:0034199 | activation of protein kinase A activity | 18/18862 | 0.001425039 | 0.003851229 | 2 |
| BP | GO:0006261 | DNA-dependent DNA replication | 157/18862 | 0.001473154 | 0.00395902 | 4 |
| BP | GO:0031100 | animal organ regeneration | 73/18862 | 0.001547741 | 0.004136362 | 3 |
| BP | GO:0009148 | pyrimidine nucleoside triphosphate biosynthetic process | 19/18862 | 0.001589492 | 0.004224473 | 2 |
| BP | GO:0000723 | telomere maintenance | 161/18862 | 0.001615661 | 0.00427043 | 4 |
| BP | GO:0000082 | G1/S transition of mitotic cell cycle | 275/18862 | 0.001672868 | 0.004373748 | 5 |
| BP | GO:0009064 | glutamine family amino acid metabolic process | 75/18862 | 0.001672935 | 0.004373748 | 3 |
| BP | GO:0010226 | response to lithium ion | 20/18862 | 0.001762557 | 0.00455851 | 2 |
| BP | GO:0032793 | positive regulation of CREB transcription factor activity | 20/18862 | 0.001762557 | 0.00455851 | 2 |
| BP | GO:0006260 | DNA replication | 280/18862 | 0.001810201 | 0.004656696 | 5 |
| BP | GO:0032200 | telomere organization | 174/18862 | 0.002144907 | 0.005488368 | 4 |
| BP | GO:0009219 | pyrimidine deoxyribonucleotide metabolic process | 23/18862 | 0.002332884 | 0.005875709 | 2 |
| BP | GO:1900273 | positive regulation of long-term synaptic potentiation | 23/18862 | 0.002332884 | 0.005875709 | 2 |
| BP | GO:0043434 | response to peptide hormone | 435/18862 | 0.002332926 | 0.005875709 | 6 |
| BP | GO:0044843 | cell cycle G1/S phase transition | 298/18862 | 0.002373803 | 0.005947521 | 5 |
| BP | GO:0007589 | body fluid secretion | 86/18862 | 0.002474568 | 0.006167862 | 3 |
| BP | GO:0034035 | purine ribonucleoside bisphosphate metabolic process | 25/18862 | 0.002755181 | 0.006796856 | 2 |
| BP | GO:0050427 | 3'-phosphoadenosine 5'-phosphosulfate metabolic process | 25/18862 | 0.002755181 | 0.006796856 | 2 |
| BP | GO:0009264 | deoxyribonucleotide catabolic process | 26/18862 | 0.002978795 | 0.007273893 | 2 |
| BP | GO:0071377 | cellular response to glucagon stimulus | 26/18862 | 0.002978795 | 0.007273893 | 2 |
| BP | GO:0022400 | regulation of rhodopsin mediated signaling pathway | 27/18862 | 0.00321065 | 0.007761263 | 2 |
| BP | GO:0046386 | deoxyribose phosphate catabolic process | 27/18862 | 0.00321065 | 0.007761263 | 2 |
| BP | GO:0016056 | rhodopsin mediated signaling pathway | 30/18862 | 0.003955141 | 0.009513155 | 2 |
| BP | GO:0007616 | long-term memory | 32/18862 | 0.004491726 | 0.010750033 | 2 |
| BP | GO:0009303 | rRNA transcription | 33/18862 | 0.004771943 | 0.011308156 | 2 |
| BP | GO:0032728 | positive regulation of interferon-beta production | 33/18862 | 0.004771943 | 0.011308156 | 2 |
| BP | GO:0042493 | response to drug | 359/18862 | 0.005244056 | 0.012366014 | 5 |
| BP | GO:0007603 | phototransduction, visible light | 35/18862 | 0.005355972 | 0.012568315 | 2 |
| BP | GO:0032869 | cellular response to insulin stimulus | 226/18862 | 0.005457567 | 0.012744549 | 4 |
| BP | GO:0033762 | response to glucagon | 37/18862 | 0.005971128 | 0.013876458 | 2 |
| BP | GO:0003091 | renal water homeostasis | 38/18862 | 0.006290254 | 0.014478196 | 2 |
| BP | GO:0007223 | Wnt signaling pathway, calcium modulating pathway | 38/18862 | 0.006290254 | 0.014478196 | 2 |
| BP | GO:0007190 | activation of adenylate cyclase activity | 39/18862 | 0.006617012 | 0.015157766 | 2 |
| BP | GO:0014075 | response to amine | 42/18862 | 0.007642591 | 0.017424116 | 2 |
| BP | GO:0046683 | response to organophosphorus | 130/18862 | 0.007851271 | 0.017815445 | 3 |
| BP | GO:0030879 | mammary gland development | 132/18862 | 0.008186096 | 0.018487996 | 3 |
| BP | GO:0150076 | neuroinflammatory response | 44/18862 | 0.008363577 | 0.018800565 | 2 |
| BP | GO:1900271 | regulation of long-term synaptic potentiation | 45/18862 | 0.008735105 | 0.019544397 | 2 |
| BP | GO:0014074 | response to purine-containing compound | 144/18862 | 0.010371297 | 0.02309787 | 3 |
| BP | GO:0032648 | regulation of interferon-beta production | 50/18862 | 0.010701189 | 0.023722742 | 2 |
| BP | GO:0032868 | response to insulin | 278/18862 | 0.011142375 | 0.024587472 | 4 |
| BP | GO:0032608 | interferon-beta production | 52/18862 | 0.011537348 | 0.025342793 | 2 |
| BP | GO:0000731 | DNA synthesis involved in DNA repair | 53/18862 | 0.011965897 | 0.026164664 | 2 |
| BP | GO:0007602 | phototransduction | 59/18862 | 0.014680859 | 0.031955953 | 2 |
| BP | GO:0019933 | cAMP-mediated signaling | 62/18862 | 0.01612866 | 0.034792531 | 2 |
| BP | GO:0046365 | monosaccharide catabolic process | 62/18862 | 0.01612866 | 0.034792531 | 2 |
| BP | GO:0035690 | cellular response to drug | 63/18862 | 0.016624343 | 0.035701715 | 2 |
| BP | GO:0009584 | detection of visible light | 66/18862 | 0.018150024 | 0.038804963 | 2 |
| BP | GO:0009749 | response to glucose | 185/18862 | 0.020198615 | 0.042993791 | 3 |
| BP | GO:0050891 | multicellular organismal water homeostasis | 72/18862 | 0.021371317 | 0.045289552 | 2 |
| BP | GO:0009746 | response to hexose | 190/18862 | 0.021652905 | 0.04568503 | 3 |
| BP | GO:1901605 | alpha-amino acid metabolic process | 191/18862 | 0.021950501 | 0.046110683 | 3 |
| BP | GO:0031099 | regeneration | 192/18862 | 0.022250345 | 0.046537334 | 3 |
| BP | GO:1901654 | response to ketone | 193/18862 | 0.022552436 | 0.046964972 | 3 |
| BP | GO:0071897 | DNA biosynthetic process | 194/18862 | 0.022856774 | 0.047393584 | 3 |
| BP | GO:0034284 | response to monosaccharide | 196/18862 | 0.023472195 | 0.048460777 | 3 |
| BP | GO:0009583 | detection of light stimulus | 77/18862 | 0.024223333 | 0.049797856 | 2 |
| BP | GO:0030104 | water homeostasis | 79/18862 | 0.025405533 | 0.052005952 | 2 |
| BP | GO:0071277 | cellular response to calcium ion | 82/18862 | 0.027222155 | 0.055488515 | 2 |
| BP | GO:0060291 | long-term synaptic potentiation | 83/18862 | 0.027839086 | 0.056506607 | 2 |
| BP | GO:0007193 | adenylate cyclase-inhibiting G protein-coupled receptor signaling pathway | 85/18862 | 0.029089793 | 0.058797149 | 2 |
| BP | GO:0007601 | visual perception | 215/18862 | 0.02976669 | 0.059913576 | 3 |
| BP | GO:0071241 | cellular response to inorganic substance | 216/18862 | 0.030120386 | 0.060372879 | 3 |
| BP | GO:0009176 | pyrimidine deoxyribonucleoside monophosphate metabolic process | 10/18862 | 0.03085044 | 0.061325145 | 1 |
| BP | GO:0009396 | folic acid-containing compound biosynthetic process | 10/18862 | 0.03085044 | 0.061325145 | 1 |
| BP | GO:0050953 | sensory perception of light stimulus | 219/18862 | 0.031194869 | 0.061754624 | 3 |
| BP | GO:0019935 | cyclic-nucleotide-mediated signaling | 89/18862 | 0.031657472 | 0.062158817 | 2 |
| BP | GO:0097306 | cellular response to alcohol | 89/18862 | 0.031657472 | 0.062158817 | 2 |
| BP | GO:0009743 | response to carbohydrate | 221/18862 | 0.031922338 | 0.062424085 | 3 |
| BP | GO:1901655 | cellular response to ketone | 92/18862 | 0.033639977 | 0.065460889 | 2 |
| BP | GO:0008655 | pyrimidine-containing compound salvage | 11/18862 | 0.03388353 | 0.065460889 | 1 |
| BP | GO:0043097 | pyrimidine nucleoside salvage | 11/18862 | 0.03388353 | 0.065460889 | 1 |
| BP | GO:0051591 | response to cAMP | 93/18862 | 0.034311414 | 0.066022385 | 2 |
| BP | GO:0021537 | telencephalon development | 231/18862 | 0.035692952 | 0.068407126 | 3 |
| BP | GO:0031953 | negative regulation of protein autophosphorylation | 12/18862 | 0.036907289 | 0.069899009 | 1 |
| BP | GO:0038003 | opioid receptor signaling pathway | 12/18862 | 0.036907289 | 0.069899009 | 1 |
| BP | GO:0071872 | cellular response to epinephrine stimulus | 12/18862 | 0.036907289 | 0.069899009 | 1 |
| BP | GO:0055062 | phosphate ion homeostasis | 13/18862 | 0.039921743 | 0.07443589 | 1 |
| BP | GO:0060213 | positive regulation of nuclear-transcribed mRNA poly(A) tail shortening | 13/18862 | 0.039921743 | 0.07443589 | 1 |
| BP | GO:0072506 | trivalent inorganic anion homeostasis | 13/18862 | 0.039921743 | 0.07443589 | 1 |
| BP | GO:1900452 | regulation of long-term synaptic depression | 13/18862 | 0.039921743 | 0.07443589 | 1 |
| BP | GO:0009143 | nucleoside triphosphate catabolic process | 14/18862 | 0.042926922 | 0.077924939 | 1 |
| BP | GO:0009173 | pyrimidine ribonucleoside monophosphate metabolic process | 14/18862 | 0.042926922 | 0.077924939 | 1 |
| BP | GO:0030002 | cellular anion homeostasis | 14/18862 | 0.042926922 | 0.077924939 | 1 |
| BP | GO:0043951 | negative regulation of cAMP-mediated signaling | 14/18862 | 0.042926922 | 0.077924939 | 1 |
| BP | GO:0046049 | UMP metabolic process | 14/18862 | 0.042926922 | 0.077924939 | 1 |
| BP | GO:0046325 | negative regulation of glucose import | 14/18862 | 0.042926922 | 0.077924939 | 1 |
| BP | GO:0046415 | urate metabolic process | 14/18862 | 0.042926922 | 0.077924939 | 1 |
| BP | GO:0007613 | memory | 107/18862 | 0.044244316 | 0.080014453 | 2 |
| BP | GO:0019321 | pentose metabolic process | 15/18862 | 0.045922854 | 0.080920548 | 1 |
| BP | GO:0042053 | regulation of dopamine metabolic process | 15/18862 | 0.045922854 | 0.080920548 | 1 |
| BP | GO:0042069 | regulation of catecholamine metabolic process | 15/18862 | 0.045922854 | 0.080920548 | 1 |
| BP | GO:0042559 | pteridine-containing compound biosynthetic process | 15/18862 | 0.045922854 | 0.080920548 | 1 |
| BP | GO:0043117 | positive regulation of vascular permeability | 15/18862 | 0.045922854 | 0.080920548 | 1 |
| BP | GO:0060211 | regulation of nuclear-transcribed mRNA poly(A) tail shortening | 15/18862 | 0.045922854 | 0.080920548 | 1 |
| BP | GO:0071871 | response to epinephrine | 15/18862 | 0.045922854 | 0.080920548 | 1 |
| BP | GO:0051607 | defense response to virus | 260/18862 | 0.047866231 | 0.08373155 | 3 |
| BP | GO:0140546 | defense response to symbiont | 260/18862 | 0.047866231 | 0.08373155 | 3 |
| BP | GO:0046134 | pyrimidine nucleoside biosynthetic process | 16/18862 | 0.048909565 | 0.084938899 | 1 |
| BP | GO:1902969 | mitotic DNA replication | 16/18862 | 0.048909565 | 0.084938899 | 1 |
| CC | GO:0061695 | transferase complex, transferring phosphorus-containing groups | 253/19520 | 1.09E-26 | 1.19E-24 | 22 |
| CC | GO:0055029 | nuclear DNA-directed RNA polymerase complex | 103/19520 | 1.54E-25 | 6.68E-24 | 17 |
| CC | GO:0000428 | DNA-directed RNA polymerase complex | 104/19520 | 1.83E-25 | 6.68E-24 | 17 |
| CC | GO:0030880 | RNA polymerase complex | 108/19520 | 3.64E-25 | 9.97E-24 | 17 |
| CC | GO:0005665 | RNA polymerase II, core complex | 15/19520 | 3.10E-17 | 6.79E-16 | 8 |
| CC | GO:0005666 | RNA polymerase III complex | 18/19520 | 2.09E-16 | 3.82E-15 | 8 |
| CC | GO:0005736 | RNA polymerase I complex | 13/19520 | 3.05E-15 | 4.77E-14 | 7 |
| CC | GO:0016591 | RNA polymerase II, holoenzyme | 79/19520 | 1.08E-10 | 1.48E-09 | 8 |
| CC | GO:0042575 | DNA polymerase complex | 20/19520 | 3.77E-07 | 4.58E-06 | 4 |
| CC | GO:0042622 | photoreceptor outer segment membrane | 16/19520 | 0.001084442 | 0.01187179 | 2 |
| CC | GO:0043601 | nuclear replisome | 22/19520 | 0.002062946 | 0.020530755 | 2 |
| CC | GO:0097381 | photoreceptor disc membrane | 23/19520 | 0.00225496 | 0.020571564 | 2 |
| CC | GO:0030894 | replisome | 24/19520 | 0.002455105 | 0.020674564 | 2 |
| CC | GO:0098686 | hippocampal mossy fiber to CA3 synapse | 30/19520 | 0.003823927 | 0.029901385 | 2 |
| CC | GO:0043596 | nuclear replication fork | 35/19520 | 0.005179151 | 0.037798716 | 2 |
| CC | GO:0000228 | nuclear chromosome | 250/19520 | 0.007308556 | 0.050005913 | 4 |
| CC | GO:0005657 | replication fork | 65/19520 | 0.017069862 | 0.109923569 | 2 |
| CC | GO:0098685 | Schaffer collateral - CA1 synapse | 67/19520 | 0.018074089 | 0.109924282 | 2 |
| CC | GO:0060170 | ciliary membrane | 72/19520 | 0.020691134 | 0.119217615 | 2 |
| CC | GO:0001750 | photoreceptor outer segment | 89/19520 | 0.030666996 | 0.167861453 | 2 |
| CC | GO:0031010 | ISWI-type complex | 11/19520 | 0.033304995 | 0.173620026 | 1 |
| CC | GO:0045178 | basal part of cell | 258/19520 | 0.045016463 | 0.214870414 | 3 |
| CC | GO:0005671 | Ada2/Gcn5/Ada3 transcription activator complex | 15/19520 | 0.045143448 | 0.214870414 | 1 |
| CC | GO:0097733 | photoreceptor cell cilium | 115/19520 | 0.048808173 | 0.222633772 | 2 |
| MF | GO:0003899 | DNA-directed 5'-3' RNA polymerase activity | 39/18337 | 6.90E-31 | 2.73E-29 | 16 |
| MF | GO:0016779 | nucleotidyltransferase activity | 131/18337 | 1.02E-30 | 2.73E-29 | 21 |
| MF | GO:0034062 | 5'-3' RNA polymerase activity | 43/18337 | 4.81E-30 | 6.46E-29 | 16 |
| MF | GO:0097747 | RNA polymerase activity | 43/18337 | 4.81E-30 | 6.46E-29 | 16 |
| MF | GO:0019205 | nucleobase-containing compound kinase activity | 42/18337 | 6.91E-19 | 7.41E-18 | 11 |
| MF | GO:0140098 | catalytic activity, acting on RNA | 386/18337 | 7.02E-14 | 6.28E-13 | 16 |
| MF | GO:0016776 | phosphotransferase activity, phosphate group as acceptor | 39/18337 | 4.60E-13 | 3.53E-12 | 8 |
| MF | GO:0050145 | nucleoside monophosphate kinase activity | 20/18337 | 4.72E-09 | 3.17E-08 | 5 |
| MF | GO:0000287 | magnesium ion binding | 216/18337 | 3.25E-08 | 1.94E-07 | 9 |
| MF | GO:0008253 | 5'-nucleotidase activity | 13/18337 | 7.24E-08 | 3.89E-07 | 4 |
| MF | GO:0008252 | nucleotidase activity | 15/18337 | 1.38E-07 | 6.71E-07 | 4 |
| MF | GO:0004550 | nucleoside diphosphate kinase activity | 18/18337 | 3.06E-07 | 1.37E-06 | 4 |
| MF | GO:0004016 | adenylate cyclase activity | 10/18337 | 3.93E-06 | 1.62E-05 | 3 |
| MF | GO:0017110 | nucleoside-diphosphatase activity | 14/18337 | 1.18E-05 | 4.53E-05 | 3 |
| MF | GO:0042578 | phosphoric ester hydrolase activity | 367/18337 | 2.46E-05 | 8.81E-05 | 8 |
| MF | GO:0009975 | cyclase activity | 22/18337 | 4.91E-05 | 0.00015495 | 3 |
| MF | GO:0016849 | phosphorus-oxygen lyase activity | 22/18337 | 4.91E-05 | 0.00015495 | 3 |
| MF | GO:0004114 | 3',5'-cyclic-nucleotide phosphodiesterase activity | 23/18337 | 5.63E-05 | 0.000167902 | 3 |
| MF | GO:0003887 | DNA-directed DNA polymerase activity | 25/18337 | 7.28E-05 | 0.000195337 | 3 |
| MF | GO:0004112 | cyclic-nucleotide phosphodiesterase activity | 25/18337 | 7.28E-05 | 0.000195337 | 3 |
| MF | GO:0008081 | phosphoric diester hydrolase activity | 89/18337 | 0.000205456 | 0.000525225 | 4 |
| MF | GO:0034061 | DNA polymerase activity | 36/18337 | 0.000220201 | 0.000537333 | 3 |
| MF | GO:0051539 | 4 iron, 4 sulfur cluster binding | 42/18337 | 0.000349144 | 0.000814936 | 3 |
| MF | GO:0016884 | carbon-nitrogen ligase activity, with glutamine as amido-N-donor | 10/18337 | 0.000465858 | 0.001042051 | 2 |
| MF | GO:0003697 | single-stranded DNA binding | 116/18337 | 0.000564218 | 0.001208151 | 4 |
| MF | GO:0016763 | transferase activity, transferring pentosyl groups | 50/18337 | 0.000585124 | 0.001208151 | 3 |
| MF | GO:0047555 | 3',5'-cyclic-GMP phosphodiesterase activity | 14/18337 | 0.000934164 | 0.001857402 | 2 |
| MF | GO:0016208 | AMP binding | 15/18337 | 0.001075615 | 0.002062268 | 2 |
| MF | GO:0051536 | iron-sulfur cluster binding | 67/18337 | 0.001374823 | 0.00246021 | 3 |
| MF | GO:0051540 | metal cluster binding | 67/18337 | 0.001374823 | 0.00246021 | 3 |
| MF | GO:0032549 | ribonucleoside binding | 384/18337 | 0.001565171 | 0.002710482 | 6 |
| MF | GO:0001882 | nucleoside binding | 390/18337 | 0.001693042 | 0.002840301 | 6 |
| MF | GO:0004551 | nucleotide diphosphatase activity | 20/18337 | 0.001925982 | 0.003133176 | 2 |
| MF | GO:0016829 | lyase activity | 194/18337 | 0.003731642 | 0.005892066 | 4 |
| MF | GO:0140097 | catalytic activity, acting on DNA | 204/18337 | 0.004460532 | 0.006841718 | 4 |
| MF | GO:0016879 | ligase activity, forming carbon-nitrogen bonds | 47/18337 | 0.010354825 | 0.015441406 | 2 |
| MF | GO:0016791 | phosphatase activity | 276/18337 | 0.012663315 | 0.018373515 | 4 |
| MF | GO:0004536 | deoxyribonuclease activity | 59/18337 | 0.015984214 | 0.022581577 | 2 |
| MF | GO:0004527 | exonuclease activity | 82/18337 | 0.029576776 | 0.040187083 | 2 |
| MF | GO:0004518 | nuclease activity | 206/18337 | 0.029943317 | 0.040187083 | 3 |
| MF | GO:0008296 | 3'-5'-exodeoxyribonuclease activity | 11/18337 | 0.03541919 | 0.044219797 | 1 |
| MF | GO:0030955 | potassium ion binding | 11/18337 | 0.03541919 | 0.044219797 | 1 |
| MF | GO:0047429 | nucleoside-triphosphate diphosphatase activity | 11/18337 | 0.03541919 | 0.044219797 | 1 |
| MF | GO:0019001 | guanyl nucleotide binding | 398/18337 | 0.041184336 | 0.046666307 | 4 |
| MF | GO:0032561 | guanyl ribonucleotide binding | 398/18337 | 0.041184336 | 0.046666307 | 4 |
| MF | GO:0004115 | 3',5'-cyclic-AMP phosphodiesterase activity | 13/18337 | 0.041725169 | 0.046666307 | 1 |
| MF | GO:0005095 | GTPase inhibitor activity | 13/18337 | 0.041725169 | 0.046666307 | 1 |
| MF | GO:0016840 | carbon-nitrogen lyase activity | 13/18337 | 0.041725169 | 0.046666307 | 1 |
| MF | GO:0019215 | intermediate filament binding | 15/18337 | 0.047990604 | 0.051526754 | 1 |
| MF | GO:0030553 | cGMP binding | 15/18337 | 0.047990604 | 0.051526754 | 1 |

**Table S3b. Analysis of KEGG.**

| ID | Description | BgRatio | pvalue | qvalue | Count |
| --- | --- | --- | --- | --- | --- |
| hsa00230 | Purine metabolism | 162/5894 | 3.01E-89 | 9.19E-88 | 55 |
| hsa00240 | Pyrimidine metabolism | 99/5894 | 1.34E-34 | 2.04E-33 | 27 |
| hsa03020 | RNA polymerase | 29/5894 | 5.90E-22 | 6.01E-21 | 14 |
| hsa00760 | Nicotinate and nicotinamide metabolism | 24/5894 | 2.39E-06 | 1.82E-05 | 5 |
| hsa05016 | Huntington's disease | 183/5894 | 0.000293749 | 0.001747598 | 8 |
| hsa03030 | DNA replication | 36/5894 | 0.000343493 | 0.001747598 | 4 |
| hsa04623 | Cytosolic DNA-sensing pathway | 56/5894 | 0.001860555 | 0.007715085 | 4 |
| hsa00030 | Pentose phosphate pathway | 27/5894 | 0.002021884 | 0.007715085 | 3 |
| hsa00983 | Drug metabolism - other enzymes | 52/5894 | 0.012924175 | 0.043836383 | 3 |
| hsa04744 | Phototransduction | 29/5894 | 0.030554621 | 0.093272002 | 2 |
| hsa00250 | Alanine, aspartate and glutamate metabolism | 32/5894 | 0.036660259 | 0.101736604 | 2 |
| hsa03410 | Base excision repair | 34/5894 | 0.040969543 | 0.104220767 | 2 |
| hsa04914 | Progesterone-mediated oocyte maturation | 87/5894 | 0.049177475 | 0.115477472 | 3 |

# Appendix 4

**LASSO and** **SVM-RFE genes**

**Table S4a. LASSO genes.**

| PRPS2 | POLR2E | PDE3A | AK5 |
| --- | --- | --- | --- |
| PFAS | POLR2F | PDE6A | NTPCR |
| ATIC | POLR3B | ADSS | PRIM1 |
| NT5C1A | CECR1 |  |  |

**Table S4b. SVM-RFE genes.**

| NT5C1A | POLR2E | POLR2F | NTPCR |
| --- | --- | --- | --- |
| PFAS | PRPS2 | PDE3A | NT5C |
| POLR3B | ADSS | AK3 | ATIC |

**Table S4c. InterGenes.**

| PRPS2 | NT5C1A | POLR3B | NTPCR | ATIC |
| --- | --- | --- | --- | --- |
| PFAS | POLR2E | PDE3A | ADSS | POLR2F |

# Appendix 5

**GSEA analysis**

**Table 5a. PFAS of GSEA analysis.**

| ID | setSize | NES | pvalue | qvalues | rank |
| --- | --- | --- | --- | --- | --- |
| KEGG_UBIQUITIN_MEDIATED_PROTEOLYSIS | 125 | 2.483461148 | 1.00E-10 | 1.41E-08 | 3284 |
| KEGG_SPLICEOSOME | 115 | 2.236587545 | 2.21E-09 | 1.56E-07 | 6529 |
| KEGG_NEUROACTIVE_LIGAND_RECEPTOR_INTERACTION | 262 | -1.907206413 | 5.77E-08 | 2.72E-06 | 6150 |
| KEGG_RNA_DEGRADATION | 54 | 2.121139464 | 7.61E-06 | 0.000268414 | 6653 |
| KEGG_OLFACTORY_TRANSDUCTION | 108 | -1.9420247 | 1.06E-05 | 0.000299791 | 5523 |
| KEGG_BASAL_TRANSCRIPTION_FACTORS | 32 | 1.992466372 | 0.000192557 | 0.004526783 | 6676 |
| KEGG_FOCAL_ADHESION | 194 | 1.578493817 | 0.000543169 | 0.010875372 | 4986 |
| KEGG_CELL_CYCLE | 122 | 1.690220788 | 0.000622588 | 0.010875372 | 5804 |
| KEGG_ENDOCYTOSIS | 170 | 1.656727191 | 0.00072847 | 0.010875372 | 4066 |
| KEGG_MATURITY_ONSET_DIABETES_OF_THE_YOUNG | 23 | -1.999713171 | 0.000813334 | 0.010875372 | 2500 |
| KEGG_ADHERENS_JUNCTION | 66 | 1.788023516 | 0.000848117 | 0.010875372 | 7578 |
| KEGG_PROTEIN_EXPORT | 22 | 1.980153258 | 0.000938163 | 0.011027437 | 5522 |
| KEGG_RENAL_CELL_CARCINOMA | 66 | 1.767275208 | 0.001016335 | 0.011027437 | 6840 |
| KEGG_TGF_BETA_SIGNALING_PATHWAY | 81 | 1.744257092 | 0.001107411 | 0.011157376 | 3213 |
| KEGG_PANCREATIC_CANCER | 69 | 1.757331561 | 0.001523695 | 0.014328084 | 7337 |
| KEGG_CHRONIC_MYELOID_LEUKEMIA | 72 | 1.711162058 | 0.001663715 | 0.014666964 | 6603 |
| KEGG_COLORECTAL_CANCER | 62 | 1.678052085 | 0.002281799 | 0.018932571 | 6493 |
| KEGG_SMALL_CELL_LUNG_CANCER | 84 | 1.621149726 | 0.003113357 | 0.024397065 | 4775 |
| KEGG_PATHOGENIC_ESCHERICHIA_COLI_INFECTION | 51 | 1.653129247 | 0.005612242 | 0.041664292 | 6875 |
| KEGG_ENDOMETRIAL_CANCER | 52 | 1.691313646 | 0.006782501 | 0.046344507 | 6323 |
| KEGG_VALINE_LEUCINE_AND_ISOLEUCINE_DEGRADATION | 43 | 1.66984127 | 0.007030117 | 0.046344507 | 5280 |
| KEGG_PROSTATE_CANCER | 89 | 1.56002299 | 0.00726077 | 0.046344507 | 5703 |
| KEGG_PEROXISOME | 77 | 1.58509985 | 0.00764154 | 0.046344507 | 3915 |
| KEGG_FATTY_ACID_METABOLISM | 40 | 1.646308697 | 0.008128108 | 0.046344507 | 7281 |
| KEGG_EPITHELIAL_CELL_SIGNALING_IN_HELICOBACTER_PYLORI_INFECTION | 67 | 1.551093287 | 0.008214045 | 0.046344507 | 7863 |
| KEGG_ASTHMA | 26 | -1.705514097 | 0.008705661 | 0.04722909 | 3401 |
| KEGG_CALCIUM_SIGNALING_PATHWAY | 172 | -1.401678295 | 0.009616479 | 0.050238134 | 6065 |
| KEGG_NON_SMALL_CELL_LUNG_CANCER | 54 | 1.586598934 | 0.011050881 | 0.055202872 | 6481 |
| KEGG_TASTE_TRANSDUCTION | 44 | -1.532866239 | 0.011349546 | 0.055202872 | 3065 |
| KEGG_PATHWAYS_IN_CANCER | 318 | 1.340443104 | 0.012730697 | 0.05985661 | 6721 |
| KEGG_N_GLYCAN_BIOSYNTHESIS | 44 | 1.548961493 | 0.01418698 | 0.062477316 | 6586 |
| KEGG_GLYCOSPHINGOLIPID_BIOSYNTHESIS_LACTO_AND_NEOLACTO_SERIES | 25 | -1.701214116 | 0.014227678 | 0.062477316 | 6132 |
| KEGG_PROTEASOME | 40 | 1.579327299 | 0.014616894 | 0.062477316 | 5773 |
| KEGG_OOCYTE_MEIOSIS | 107 | 1.476309167 | 0.016427889 | 0.068152851 | 3909 |
| KEGG_WNT_SIGNALING_PATHWAY | 146 | 1.394486969 | 0.020166157 | 0.07415271 | 6527 |
| KEGG_MTOR_SIGNALING_PATHWAY | 50 | 1.507982425 | 0.020327074 | 0.07415271 | 4437 |
| KEGG_APOPTOSIS | 87 | 1.455636036 | 0.020371579 | 0.07415271 | 4645 |
| KEGG_FC_GAMMA_R_MEDIATED_PHAGOCYTOSIS | 90 | 1.463964096 | 0.020498539 | 0.07415271 | 3999 |
| KEGG_PROPANOATE_METABOLISM | 31 | 1.560110394 | 0.020502671 | 0.07415271 | 5031 |
| KEGG_ERBB_SIGNALING_PATHWAY | 86 | 1.510781659 | 0.023080594 | 0.080371506 | 6522 |
| KEGG_INOSITOL_PHOSPHATE_METABOLISM | 54 | 1.506552949 | 0.023361718 | 0.080371506 | 3542 |
| KEGG_NUCLEOTIDE_EXCISION_REPAIR | 43 | 1.496711016 | 0.027921597 | 0.09377178 | 5711 |
| KEGG_NEUROTROPHIN_SIGNALING_PATHWAY | 122 | 1.389625296 | 0.033616497 | 0.110271985 | 6534 |
| KEGG_CITRATE_CYCLE_TCA_CYCLE | 30 | 1.527665951 | 0.037155967 | 0.11911243 | 7227 |
| KEGG_ALZHEIMERS_DISEASE | 138 | 1.338656756 | 0.038095238 | 0.119409635 | 5589 |
| KEGG_ETHER_LIPID_METABOLISM | 25 | 1.5107599 | 0.041186161 | 0.126291662 | 3651 |
| KEGG_REGULATION_OF_ACTIN_CYTOSKELETON | 208 | 1.340788559 | 0.042365082 | 0.127142687 | 4870 |
| KEGG_THYROID_CANCER | 29 | 1.485485443 | 0.045378151 | 0.133348076 | 6045 |
| KEGG_GLYCOSYLPHOSPHATIDYLINOSITOL_GPI_ANCHOR_BIOSYNTHESIS | 24 | 1.57231456 | 0.046998478 | 0.135291001 | 4716 |
| KEGG_PHOSPHATIDYLINOSITOL_SIGNALING_SYSTEM | 74 | 1.384431304 | 0.049107143 | 0.138533835 | 6336 |

**Table 5b. POLR2F of GSEA analysis.**

| ID | setSize | NES | pvalue | qvalues | rank |
| --- | --- | --- | --- | --- | --- |
| KEGG_UBIQUITIN_MEDIATED_PROTEOLYSIS | 125 | -2.329110646 | 1.00E-10 | 1.44E-08 | 4806 |
| KEGG_SPLICEOSOME | 115 | -2.202974628 | 5.85E-09 | 4.22E-07 | 5870 |
| KEGG_OLFACTORY_TRANSDUCTION | 108 | 2.097906176 | 1.25E-06 | 6.01E-05 | 8619 |
| KEGG_RNA_DEGRADATION | 54 | -2.13934644 | 3.15E-06 | 0.000113509 | 4917 |
| KEGG_NEUROACTIVE_LIGAND_RECEPTOR_INTERACTION | 262 | 1.57429355 | 2.94E-05 | 0.00084697 | 5810 |
| KEGG_PROTEIN_EXPORT | 22 | -1.99501012 | 0.000336553 | 0.007309114 | 6181 |
| KEGG_CITRATE_CYCLE_TCA_CYCLE | 30 | -1.960987718 | 0.000354785 | 0.007309114 | 3625 |
| KEGG_MATURITY_ONSET_DIABETES_OF_THE_YOUNG | 23 | 2.104570528 | 0.000554982 | 0.009637056 | 6351 |
| KEGG_PRIMARY_IMMUNODEFICIENCY | 35 | 2.003584068 | 0.000614509 | 0.009637056 | 5162 |
| KEGG_PROPANOATE_METABOLISM | 31 | -1.867695092 | 0.000668263 | 0.009637056 | 4621 |
| KEGG_SMALL_CELL_LUNG_CANCER | 84 | -1.763070917 | 0.000738252 | 0.009678518 | 4699 |
| KEGG_FATTY_ACID_METABOLISM | 40 | -1.862077459 | 0.000898701 | 0.010800182 | 4867 |
| KEGG_VALINE_LEUCINE_AND_ISOLEUCINE_DEGRADATION | 43 | -1.82930461 | 0.000990966 | 0.010992898 | 4894 |
| KEGG_BASAL_TRANSCRIPTION_FACTORS | 32 | -1.816788418 | 0.001354516 | 0.013952532 | 5470 |
| KEGG_ADIPOCYTOKINE_SIGNALING_PATHWAY | 65 | -1.734491363 | 0.001577056 | 0.01516187 | 4874 |
| KEGG_INSULIN_SIGNALING_PATHWAY | 133 | -1.56823117 | 0.001886794 | 0.017005971 | 4654 |
| KEGG_COLORECTAL_CANCER | 62 | -1.709938125 | 0.002024768 | 0.01717605 | 4590 |
| KEGG_PEROXISOME | 77 | -1.67164419 | 0.002268065 | 0.018171049 | 4007 |
| KEGG_FOCAL_ADHESION | 194 | -1.488189928 | 0.002696137 | 0.019398557 | 5396 |
| KEGG_CELL_CYCLE | 122 | -1.580112206 | 0.002782102 | 0.019398557 | 5731 |
| KEGG_NICOTINATE_AND_NICOTINAMIDE_METABOLISM | 23 | -1.782288702 | 0.002824826 | 0.019398557 | 3786 |
| KEGG_TGF_BETA_SIGNALING_PATHWAY | 81 | -1.654449057 | 0.003160406 | 0.02071654 | 6912 |
| KEGG_ENDOCYTOSIS | 170 | -1.517183033 | 0.00406483 | 0.025486575 | 5334 |
| KEGG_PROTEASOME | 40 | -1.70987427 | 0.004727333 | 0.028405468 | 6188 |
| KEGG_ADHERENS_JUNCTION | 66 | -1.624504648 | 0.005330625 | 0.030749291 | 6158 |
| KEGG_NEUROTROPHIN_SIGNALING_PATHWAY | 122 | -1.52778476 | 0.006128471 | 0.033991922 | 5356 |
| KEGG_CHRONIC_MYELOID_LEUKEMIA | 72 | -1.589443777 | 0.006604777 | 0.03446286 | 6125 |
| KEGG_PATHWAYS_IN_CANCER | 318 | -1.401723252 | 0.006691329 | 0.03446286 | 6463 |
| KEGG_N_GLYCAN_BIOSYNTHESIS | 44 | -1.628306173 | 0.008276466 | 0.041157019 | 6352 |
| KEGG_PYRUVATE_METABOLISM | 39 | -1.613733032 | 0.009606341 | 0.046177852 | 5033 |
| KEGG_APOPTOSIS | 87 | -1.504627674 | 0.011501123 | 0.053502676 | 4274 |
| KEGG_PANCREATIC_CANCER | 69 | -1.501217741 | 0.013923689 | 0.062111531 | 4874 |
| KEGG_RENAL_CELL_CARCINOMA | 66 | -1.519845021 | 0.014960749 | 0.062111531 | 7004 |
| KEGG_HEMATOPOIETIC_CELL_LINEAGE | 83 | 1.510770629 | 0.015040096 | 0.062111531 | 4437 |
| KEGG_PROGESTERONE_MEDIATED_OOCYTE_MATURATION | 83 | -1.48531487 | 0.015074514 | 0.062111531 | 5401 |
| KEGG_CIRCADIAN_RHYTHM_MAMMAL | 11 | -1.671933467 | 0.016552902 | 0.066308408 | 2512 |
| KEGG_PROSTATE_CANCER | 89 | -1.483581561 | 0.017454168 | 0.068029046 | 6125 |
| KEGG_NUCLEOTIDE_EXCISION_REPAIR | 43 | -1.556248042 | 0.018048857 | 0.068495661 | 4354 |
| KEGG_GLYCOSYLPHOSPHATIDYLINOSITOL_GPI_ANCHOR_BIOSYNTHESIS | 24 | -1.577096428 | 0.022125001 | 0.081811745 | 5000 |
| KEGG_MTOR_SIGNALING_PATHWAY | 50 | -1.507280256 | 0.022773771 | 0.082105436 | 4040 |
| KEGG_INOSITOL_PHOSPHATE_METABOLISM | 54 | -1.488567762 | 0.034210526 | 0.119304936 | 3688 |
| KEGG_CYTOKINE_CYTOKINE_RECEPTOR_INTERACTION | 250 | 1.198568013 | 0.034746474 | 0.119304936 | 4320 |
| KEGG_WNT_SIGNALING_PATHWAY | 146 | -1.365324845 | 0.037383178 | 0.123604466 | 6961 |
| KEGG_OOCYTE_MEIOSIS | 107 | -1.407104504 | 0.037712895 | 0.123604466 | 5619 |
| KEGG_PPAR_SIGNALING_PATHWAY | 66 | -1.46264228 | 0.041290323 | 0.132322203 | 3227 |
| KEGG_LYSINE_DEGRADATION | 39 | -1.475155631 | 0.046544429 | 0.145917317 | 6983 |
| KEGG_BETA_ALANINE_METABOLISM | 22 | -1.535705427 | 0.048136646 | 0.147698107 | 4621 |

# Appendix 6

**Drug prediction**

**Table 6. Drug prediction.**

| search_term | gene | drug | interaction_types | sources |
| --- | --- | --- | --- | --- |
| ATIC | ATIC | PEMETREXED | inhibitor | DTC |
| ATIC | ATIC | METHOTREXATE | inhibitor | NCI|PharmGKB |
| NT5C1A | NT5C1A | GEMCITABINE | unknown | PharmGKB |
| NT5C1A | NT5C1A | CLADRIBINE | unknown | PharmGKB |
| NT5C1A | NT5C1A | FLUOROURACIL | unknown | PharmGKB |
| PDE3A | PDE3A | PENTOXIFYLLINE | inhibitor | ChemblInteractions |
| PDE3A | PDE3A | THEOPHYLLINE SODIUM GLYCINATE | inhibitor | ChemblInteractions |
| PDE3A | PDE3A | THEOPHYLLINE | inhibitor | TdgClinicalTrial|ChemblInteractions|TEND |
| PDE3A | PDE3A | ANAGRELIDE | inhibitor | TTD |
| PDE3A | PDE3A | AMINOPHYLLINE | inhibitor | ChemblInteractions|TTD |
| PDE3A | PDE3A | OXTRIPHYLLINE | inhibitor | ChemblInteractions|TTD |
| PDE3A | PDE3A | CILOSTAZOL | inhibitor | TdgClinicalTrial|ChemblInteractions|TEND|TTD |
| PDE3A | PDE3A | DYPHYLLINE | inhibitor | ChemblInteractions |
| PDE3A | PDE3A | MILRINONE | inhibitor | TdgClinicalTrial|TEND|TTD |
| PDE3A | PDE3A | ENOXIMONE | inhibitor | TdgClinicalTrial|TEND|TTD |
| PDE3A | PDE3A | MILRINONE LACTATE | inhibitor | ChemblInteractions |
| PDE3A | PDE3A | INAMRINONE LACTATE | inhibitor | ChemblInteractions |
| PDE3A | PDE3A | ANAGRELIDE HYDROCHLORIDE | inhibitor | ChemblInteractions |
| PDE3A | PDE3A | INAMRINONE | inhibitor | TdgClinicalTrial|TEND |
| PDE3A | PDE3A | DIPYRIDAMOLE | inhibitor | ChemblInteractions |
| PDE3A | PDE3A | HYDROCHLOROTHIAZIDE |  | PharmGKB |
| PDE3A | PDE3A | K-134 | unknown | TTD |
| PDE3A | PDE3A | VESNARINONE | unknown | TTD |
| PDE3A | PDE3A | BEMORADAN | unknown | TTD |
| PDE3A | PDE3A | OLPRINONE | unknown | TTD |
| PDE3A | PDE3A | TIPELUKAST | unknown | TTD |
| ADSS | ADSS2 | ALANOSINE | unknown | NCI |

# Appendix 7

**MiRNA and LncRNA**

**Table 7a. Gene-miRNA.**

| Gene | miRNA | miRanda | miRDB | TargetScan | Sum |
| --- | --- | --- | --- | --- | --- |
| ADSS | hsa-miR-3133 | 1 | 1 | 1 | 3 |
| POLR2F | hsa-miR-4287 | 1 | 1 | 1 | 3 |
| POLR2F | hsa-miR-126-5p | 1 | 1 | 1 | 3 |
| ADSS | hsa-miR-767-5p | 1 | 1 | 1 | 3 |
| POLR2F | hsa-miR-4261 | 1 | 1 | 1 | 3 |
| PDE3A | hsa-miR-211-5p | 1 | 1 | 1 | 3 |
| POLR2E | hsa-miR-150-5p | 1 | 1 | 1 | 3 |
| ADSS | hsa-miR-497-5p | 1 | 1 | 1 | 3 |
| PRPS2 | hsa-miR-4261 | 1 | 1 | 1 | 3 |
| PDE3A | hsa-miR-4302 | 1 | 1 | 1 | 3 |
| POLR2E | hsa-miR-185-5p | 1 | 1 | 1 | 3 |
| PRPS2 | hsa-miR-659-3p | 1 | 1 | 1 | 3 |
| POLR2E | hsa-miR-149-3p | 1 | 1 | 1 | 3 |
| ADSS | hsa-miR-214-3p | 1 | 1 | 1 | 3 |
| POLR3B | hsa-miR-1257 | 1 | 1 | 1 | 3 |
| ADSS | hsa-miR-761 | 1 | 1 | 1 | 3 |
| PDE3A | hsa-miR-1204 | 1 | 1 | 1 | 3 |
| PDE3A | hsa-miR-139-5p | 1 | 1 | 1 | 3 |
| POLR3B | hsa-miR-136-5p | 1 | 1 | 1 | 3 |
| ADSS | hsa-miR-548a-3p | 1 | 1 | 1 | 3 |
| PFAS | hsa-miR-4268 | 1 | 1 | 1 | 3 |
| PRPS2 | hsa-miR-1245a | 1 | 1 | 1 | 3 |
| POLR2F | hsa-miR-1275 | 1 | 1 | 1 | 3 |
| PRPS2 | hsa-miR-1278 | 1 | 1 | 1 | 3 |
| POLR2E | hsa-miR-1234-3p | 1 | 1 | 1 | 3 |
| POLR2F | hsa-miR-466 | 1 | 1 | 1 | 3 |
| POLR2F | hsa-miR-4271 | 1 | 1 | 1 | 3 |
| POLR2F | hsa-miR-4268 | 1 | 1 | 1 | 3 |
| PDE3A | hsa-miR-548v | 1 | 1 | 1 | 3 |
| PDE3A | hsa-miR-4307 | 1 | 1 | 1 | 3 |
| ADSS | hsa-miR-526b-5p | 1 | 1 | 1 | 3 |
| PDE3A | hsa-miR-497-3p | 1 | 1 | 1 | 3 |
| ADSS | hsa-miR-577 | 1 | 1 | 1 | 3 |
| POLR2F | hsa-miR-211-5p | 1 | 1 | 1 | 3 |
| POLR2F | hsa-miR-3179 | 1 | 1 | 1 | 3 |
| PFAS | hsa-miR-4288 | 1 | 1 | 1 | 3 |
| POLR2F | hsa-miR-3065-3p | 1 | 1 | 1 | 3 |
| ADSS | hsa-miR-3065-5p | 1 | 1 | 1 | 3 |
| PRPS2 | hsa-miR-524-5p | 1 | 1 | 1 | 3 |
| ADSS | hsa-miR-29b-2-5p | 1 | 1 | 1 | 3 |
| PDE3A | hsa-miR-2115-3p | 1 | 1 | 1 | 3 |
| PRPS2 | hsa-miR-501-5p | 1 | 1 | 1 | 3 |
| POLR2F | hsa-miR-765 | 1 | 1 | 1 | 3 |
| PDE3A | hsa-miR-138-5p | 1 | 1 | 1 | 3 |
| ADSS | hsa-miR-1323 | 1 | 1 | 1 | 3 |
| ADSS | hsa-miR-490-5p | 1 | 1 | 1 | 3 |
| ADSS | hsa-miR-34a-3p | 1 | 1 | 1 | 3 |
| POLR3B | hsa-miR-150-3p | 1 | 1 | 1 | 3 |
| PDE3A | hsa-miR-3074-3p | 1 | 1 | 1 | 3 |
| PDE3A | hsa-miR-155-5p | 1 | 1 | 1 | 3 |
| ADSS | hsa-miR-3185 | 1 | 1 | 1 | 3 |
| ADSS | hsa-miR-922 | 1 | 1 | 1 | 3 |
| PRPS2 | hsa-miR-513a-3p | 1 | 1 | 1 | 3 |
| ATIC | hsa-miR-452-5p | 1 | 1 | 1 | 3 |
| ADSS | hsa-miR-548n | 1 | 1 | 1 | 3 |
| PFAS | hsa-miR-921 | 1 | 1 | 1 | 3 |
| POLR2E | hsa-miR-561-3p | 1 | 1 | 1 | 3 |
| PRPS2 | hsa-miR-644a | 1 | 1 | 1 | 3 |
| PFAS | hsa-miR-423-5p | 1 | 1 | 1 | 3 |
| ADSS | hsa-miR-2113 | 1 | 1 | 1 | 3 |
| POLR2F | hsa-miR-149-3p | 1 | 1 | 1 | 3 |
| PDE3A | hsa-miR-29b-2-5p | 1 | 1 | 1 | 3 |
| ADSS | hsa-miR-2054 | 1 | 1 | 1 | 3 |
| PRPS2 | hsa-miR-877-5p | 1 | 1 | 1 | 3 |
| PRPS2 | hsa-miR-3179 | 1 | 1 | 1 | 3 |
| POLR2E | hsa-miR-1207-5p | 1 | 1 | 1 | 3 |
| PDE3A | hsa-miR-130b-5p | 1 | 1 | 1 | 3 |
| POLR3B | hsa-miR-3145-3p | 1 | 1 | 1 | 3 |
| POLR2F | hsa-miR-1200 | 1 | 1 | 1 | 3 |
| POLR3B | hsa-miR-590-5p | 1 | 1 | 1 | 3 |
| ADSS | hsa-miR-16-5p | 1 | 1 | 1 | 3 |
| PFAS | hsa-miR-1827 | 1 | 1 | 1 | 3 |
| POLR3B | hsa-miR-524-5p | 1 | 1 | 1 | 3 |
| PDE3A | hsa-miR-27a-3p | 1 | 1 | 1 | 3 |
| PRPS2 | hsa-miR-617 | 1 | 1 | 1 | 3 |
| PRPS2 | hsa-miR-570-3p | 1 | 1 | 1 | 3 |
| POLR2F | hsa-miR-204-5p | 1 | 1 | 1 | 3 |
| POLR2F | hsa-miR-4324 | 1 | 1 | 1 | 3 |
| ADSS | hsa-miR-4291 | 1 | 1 | 1 | 3 |
| PRPS2 | hsa-miR-4283 | 1 | 1 | 1 | 3 |
| POLR2E | hsa-miR-663b | 1 | 1 | 1 | 3 |
| ADSS | hsa-miR-20a-3p | 1 | 1 | 1 | 3 |
| POLR3B | hsa-miR-657 | 1 | 1 | 1 | 3 |
| PRPS2 | hsa-miR-9-5p | 1 | 1 | 1 | 3 |
| ADSS | hsa-miR-195-5p | 1 | 1 | 1 | 3 |
| PDE3A | hsa-miR-186-3p | 1 | 1 | 1 | 3 |
| PRPS2 | hsa-miR-21-3p | 1 | 1 | 1 | 3 |
| ADSS | hsa-miR-3143 | 1 | 1 | 1 | 3 |
| PDE3A | hsa-miR-299-3p | 1 | 1 | 1 | 3 |
| PRPS2 | hsa-miR-936 | 1 | 1 | 1 | 3 |
| POLR3B | hsa-miR-4273 | 1 | 1 | 1 | 3 |
| ADSS | hsa-miR-33a-3p | 1 | 1 | 1 | 3 |
| ADSS | hsa-miR-2115-3p | 1 | 1 | 1 | 3 |
| PFAS | hsa-miR-466 | 1 | 1 | 1 | 3 |
| PRPS2 | hsa-miR-1284 | 1 | 1 | 1 | 3 |
| PRPS2 | hsa-miR-222-5p | 1 | 1 | 1 | 3 |
| PRPS2 | hsa-miR-4277 | 1 | 1 | 1 | 3 |
| PRPS2 | hsa-miR-3133 | 1 | 1 | 1 | 3 |
| PDE3A | hsa-miR-1207-5p | 1 | 1 | 1 | 3 |
| PRPS2 | hsa-miR-33a-3p | 1 | 1 | 1 | 3 |
| ADSS | hsa-miR-514a-3p | 1 | 1 | 1 | 3 |
| ADSS | hsa-miR-875-3p | 1 | 1 | 1 | 3 |
| ADSS | hsa-miR-1179 | 1 | 1 | 1 | 3 |
| PRPS2 | hsa-miR-4307 | 1 | 1 | 1 | 3 |
| PDE3A | hsa-miR-599 | 1 | 1 | 1 | 3 |
| PFAS | hsa-miR-2278 | 1 | 1 | 1 | 3 |
| PRPS2 | hsa-miR-107 | 1 | 1 | 1 | 3 |
| PFAS | hsa-miR-558 | 1 | 1 | 1 | 3 |
| PFAS | hsa-miR-761 | 1 | 1 | 1 | 3 |
| PRPS2 | hsa-miR-224-5p | 1 | 1 | 1 | 3 |
| POLR3B | hsa-miR-513a-5p | 1 | 1 | 1 | 3 |
| POLR2E | hsa-miR-125a-3p | 1 | 1 | 1 | 3 |
| POLR2F | hsa-miR-363-5p | 1 | 1 | 1 | 3 |
| POLR3B | hsa-miR-2117 | 1 | 1 | 1 | 3 |
| PDE3A | hsa-miR-362-3p | 1 | 1 | 1 | 3 |
| ADSS | hsa-miR-302c-5p | 1 | 1 | 1 | 3 |
| ADSS | hsa-miR-1283 | 1 | 1 | 1 | 3 |
| PRPS2 | hsa-miR-133b | 1 | 1 | 1 | 3 |
| PRPS2 | hsa-miR-875-3p | 1 | 1 | 1 | 3 |
| PDE3A | hsa-miR-27b-3p | 1 | 1 | 1 | 3 |
| POLR2E | hsa-miR-129-5p | 1 | 1 | 1 | 3 |
| PFAS | hsa-miR-214-3p | 1 | 1 | 1 | 3 |
| PRPS2 | hsa-miR-1290 | 1 | 1 | 1 | 3 |
| ADSS | hsa-miR-524-5p | 1 | 1 | 1 | 3 |
| POLR2F | hsa-miR-513a-5p | 1 | 1 | 1 | 3 |
| PDE3A | hsa-miR-204-5p | 1 | 1 | 1 | 3 |
| POLR3B | hsa-miR-3123 | 1 | 1 | 1 | 3 |
| POLR2E | hsa-miR-1237-3p | 1 | 1 | 1 | 3 |
| PDE3A | hsa-miR-340-5p | 1 | 1 | 1 | 3 |
| ADSS | hsa-miR-600 | 1 | 1 | 1 | 3 |
| ADSS | hsa-miR-3163 | 1 | 1 | 1 | 3 |
| ADSS | hsa-miR-19a-3p | 1 | 1 | 1 | 3 |
| POLR2F | hsa-miR-766-3p | 1 | 1 | 1 | 3 |
| PDE3A | hsa-miR-603 | 1 | 1 | 1 | 3 |
| ADSS | hsa-miR-28-3p | 1 | 1 | 1 | 3 |
| PFAS | hsa-miR-4251 | 1 | 1 | 1 | 3 |
| POLR2F | hsa-miR-544b | 1 | 1 | 1 | 3 |
| POLR2F | hsa-miR-4251 | 1 | 1 | 1 | 3 |
| PDE3A | hsa-miR-302c-5p | 1 | 1 | 1 | 3 |
| PDE3A | hsa-miR-592 | 1 | 1 | 1 | 3 |
| PDE3A | hsa-miR-548c-3p | 1 | 1 | 1 | 3 |
| PFAS | hsa-miR-501-5p | 1 | 1 | 1 | 3 |
| PDE3A | hsa-miR-200a-5p | 1 | 1 | 1 | 3 |
| POLR2F | hsa-miR-194-3p | 1 | 1 | 1 | 3 |
| PDE3A | hsa-miR-377-3p | 1 | 1 | 1 | 3 |
| POLR2F | hsa-miR-541-5p | 1 | 1 | 1 | 3 |
| PFAS | hsa-miR-610 | 1 | 1 | 1 | 3 |
| POLR2F | hsa-miR-4286 | 1 | 1 | 1 | 3 |
| PDE3A | hsa-miR-1248 | 1 | 1 | 1 | 3 |
| POLR3B | hsa-miR-21-5p | 1 | 1 | 1 | 3 |
| ADSS | hsa-miR-148a-5p | 1 | 1 | 1 | 3 |
| PDE3A | hsa-miR-875-3p | 1 | 1 | 1 | 3 |
| PRPS2 | hsa-miR-630 | 1 | 1 | 1 | 3 |
| POLR3B | hsa-miR-1303 | 1 | 1 | 1 | 3 |
| PDE3A | hsa-miR-548p | 1 | 1 | 1 | 3 |
| POLR2E | hsa-miR-186-3p | 1 | 1 | 1 | 3 |
| PDE3A | hsa-miR-1237-3p | 1 | 1 | 1 | 3 |
| POLR2F | hsa-miR-4288 | 1 | 1 | 1 | 3 |
| POLR3B | hsa-miR-615-3p | 1 | 1 | 1 | 3 |
| POLR2F | hsa-miR-92a-1-5p | 1 | 1 | 1 | 3 |
| PFAS | hsa-miR-3145-3p | 1 | 1 | 1 | 3 |
| PDE3A | hsa-miR-224-5p | 1 | 1 | 1 | 3 |
| PDE3A | hsa-miR-502-5p | 1 | 1 | 1 | 3 |
| PFAS | hsa-miR-338-3p | 1 | 1 | 1 | 3 |
| POLR2F | hsa-miR-661 | 1 | 1 | 1 | 3 |
| ADSS | hsa-miR-4261 | 1 | 1 | 1 | 3 |
| POLR3B | hsa-miR-4320 | 1 | 1 | 1 | 3 |
| PDE3A | hsa-miR-4300 | 1 | 1 | 1 | 3 |
| POLR2E | hsa-miR-513a-5p | 1 | 1 | 1 | 3 |
| PRPS2 | hsa-miR-382-5p | 1 | 1 | 1 | 3 |
| PFAS | hsa-miR-539-5p | 1 | 1 | 1 | 3 |
| PDE3A | hsa-miR-607 | 1 | 1 | 1 | 3 |
| POLR2F | hsa-miR-1303 | 1 | 1 | 1 | 3 |
| POLR3B | hsa-miR-575 | 1 | 1 | 1 | 3 |
| POLR2F | hsa-miR-3160-3p | 1 | 1 | 1 | 3 |
| POLR2F | hsa-miR-4270 | 1 | 1 | 1 | 3 |
| ADSS | hsa-miR-19b-3p | 1 | 1 | 1 | 3 |
| PDE3A | hsa-miR-1208 | 1 | 1 | 1 | 3 |
| ADSS | hsa-miR-1305 | 1 | 1 | 1 | 3 |
| PRPS2 | hsa-miR-548c-3p | 1 | 1 | 1 | 3 |
| POLR2F | hsa-miR-4267 | 1 | 1 | 1 | 3 |
| PFAS | hsa-miR-2115-3p | 1 | 1 | 1 | 3 |
| ADSS | hsa-miR-466 | 1 | 1 | 1 | 3 |
| PFAS | hsa-miR-133b | 1 | 1 | 1 | 3 |
| PDE3A | hsa-miR-200b-5p | 1 | 1 | 1 | 3 |
| ADSS | hsa-miR-657 | 1 | 1 | 1 | 3 |
| POLR2F | hsa-miR-632 | 1 | 1 | 1 | 3 |
| ADSS | hsa-miR-659-3p | 1 | 1 | 1 | 3 |
| POLR2F | hsa-miR-1226-3p | 1 | 1 | 1 | 3 |
| PFAS | hsa-miR-609 | 1 | 1 | 1 | 3 |
| ADSS | hsa-miR-4257 | 1 | 1 | 1 | 3 |
| ADSS | hsa-miR-2116-5p | 1 | 1 | 1 | 3 |

**Table 7b. Gene-lncRNA.**

| miRNA | lncRNA |
| --- | --- |
| hsa-miR-1208 | FLJ16779 |
| hsa-miR-1200 | LINC01043 |
| hsa-miR-765 | GAS6-AS1 |
| hsa-miR-767-5p | RP11-326C3.10 |
| hsa-miR-125a-3p | RP11-10J21.4 |
| hsa-miR-570-3p | RP11-10J21.4 |
| hsa-miR-875-3p | CDR1-AS |
| hsa-miR-214-3p | LA16c-306A4.2 |
| hsa-miR-125a-3p | RP11-830F9.6 |
| hsa-miR-125a-3p | LINC00917 |
| hsa-miR-363-5p | RP11-573D15.8 |
| hsa-miR-27a-3p | RP11-10J21.4 |
| hsa-miR-92a-1-5p | MUC2 |
| hsa-miR-149-3p | C10orf91 |
| hsa-miR-423-5p | C10orf91 |
| hsa-miR-125a-3p | AC097468.4 |
| hsa-miR-136-5p | CTD-2534I21.9 |
| hsa-miR-363-5p | C10orf91 |
| hsa-miR-877-5p | TP73-AS1 |
| hsa-miR-767-5p | RP11-326C3.14 |
| hsa-miR-592 | RP11-982M15.8 |
| hsa-miR-921 | CTA-722E9.1 |
| hsa-miR-125a-3p | CH507-216K13.2 |
| hsa-miR-377-3p | LL22NC03-27C5.1 |
| hsa-miR-185-5p | CTC-265F19.1 |
| hsa-miR-766-3p | LINC01070 |
| hsa-miR-765 | RP11-138B4.1 |
| hsa-miR-138-5p | HP09025 |
| hsa-miR-423-5p | PAX8-AS1 |
| hsa-miR-185-5p | AC092657.2 |
| hsa-miR-338-3p | RP11-229P13.22 |
| hsa-miR-1237-3p | GS1-519E5.1 |
| hsa-miR-1226-3p | LINC01070 |
| hsa-miR-1234-3p | RP13-580B18.4 |
| hsa-miR-377-3p | RP4-737E23.2 |
| hsa-miR-2113 | RP11-982M15.8 |
| hsa-miR-214-3p | RP4-539M6.22 |
| hsa-miR-338-3p | RP4-539M6.22 |
| hsa-miR-125a-3p | FAM182A |
| hsa-miR-185-5p | AATBC |
| hsa-miR-765 | RP11-627G23.1 |
| hsa-miR-766-3p | RP13-507P19.2 |
| hsa-miR-136-5p | RP11-526P6.1 |
| hsa-miR-150-5p | LINC01002 |
| hsa-miR-20a-3p | LINC01043 |
| hsa-miR-1207-5p | C10orf91 |
| hsa-miR-1207-5p | RP11-618K13.2 |
| hsa-miR-92a-1-5p | RP11-13K12.1 |
| hsa-miR-423-5p | RP11-627G23.1 |
| hsa-miR-766-3p | MUC19 |
| hsa-miR-377-3p | CTD-3138B18.5 |
| hsa-miR-129-5p | RP11-166B2.5 |
| hsa-miR-592 | HPVC1 |
| hsa-miR-149-3p | CTA-941F9.10 |
| hsa-miR-766-3p | CTD-2008P7.3 |
| hsa-miR-338-3p | CTD-2292P10.2 |
| hsa-miR-363-5p | MUC2 |
| hsa-miR-1207-5p | LINC00265 |
| hsa-miR-1207-5p | RP11-333E1.2 |
| hsa-miR-377-3p | RP11-210M15.1 |
| hsa-miR-766-3p | RP13-580B18.4 |
| hsa-miR-1234-3p | LINC00174 |
| hsa-miR-558 | RP11-458F8.4 |
| hsa-miR-150-5p | LINC01165 |
| hsa-miR-149-3p | AIRN |
| hsa-miR-338-3p | LINC01165 |
| hsa-miR-766-3p | AC078942.1 |
| hsa-miR-875-3p | PCBP3-OT1 |
| hsa-miR-558 | RP11-384K6.6 |
| hsa-miR-1207-5p | AP001476.4 |
| hsa-miR-1207-5p | RP4-539M6.22 |
| hsa-miR-1234-3p | LINC01001 |
| hsa-miR-1208 | RP11-431K24.1 |
| hsa-miR-377-3p | LINC01002 |
| hsa-miR-185-5p | RP11-384K6.6 |
| hsa-miR-338-3p | RP11-780K2.1 |
| hsa-miR-299-3p | ZNF883 |
| hsa-miR-338-3p | PCBP3-OT1 |
| hsa-miR-185-5p | FLJ35934 |
| hsa-miR-1226-3p | RP4-539M6.22 |
| hsa-miR-558 | RP5-991B18.1 |
| hsa-miR-561-3p | RP11-231G3.1 |
| hsa-miR-20a-3p | RP11-54O7.17 |
| hsa-miR-663b | RP11-54O7.17 |
| hsa-miR-149-3p | LINC00265 |
| hsa-miR-1200 | LINC01123 |
| hsa-miR-149-3p | RP11-311F12.1 |
| hsa-miR-194-3p | RP4-671O14.7 |
| hsa-miR-558 | LINC00265 |
| hsa-miR-766-3p | RP11-1217F2.15 |
| hsa-miR-130b-5p | GS1-251I9.3 |
| hsa-miR-766-3p | CTD-3193O13.12 |
| hsa-miR-1208 | C22orf34 |
| hsa-miR-149-3p | LINC00689 |
| hsa-miR-2113 | AC005264.2 |
| hsa-miR-129-5p | AC006548.28 |
| hsa-miR-363-5p | AC011284.3 |
| hsa-miR-1226-3p | RP11-1217F2.15 |
| hsa-miR-185-5p | RP11-269G24.6 |
| hsa-miR-126-5p | RP11-164O23.8 |
| hsa-miR-423-5p | FLJ35934 |
| hsa-miR-27a-3p | RP11-449D8.5 |
| hsa-miR-1200 | CTD-2008P7.3 |
| hsa-miR-338-3p | MCF2L-AS1 |
| hsa-miR-766-3p | RP11-85G18.6 |
| hsa-miR-539-5p | AC018816.3 |
| hsa-miR-382-5p | MUC19 |
| hsa-miR-194-3p | MUC19 |
| hsa-miR-1207-5p | RP11-680F20.6 |
| hsa-miR-363-5p | RP11-7M8.2 |
| hsa-miR-338-3p | LINC01423 |
| hsa-miR-766-3p | LINC01022 |
| hsa-miR-1200 | RP11-22M7.2 |
| hsa-miR-1237-3p | AC091153.4 |
| hsa-miR-214-3p | TTLL10-AS1 |
| hsa-miR-224-5p | RP11-91H12.3 |
| hsa-miR-875-3p | RP11-64K12.8 |
| hsa-miR-214-3p | AC015849.16 |
| hsa-miR-1207-5p | RP11-867G23.4 |
| hsa-miR-875-3p | FRMPD3-AS1 |
| hsa-miR-214-3p | TMEM9B-AS1 |
| hsa-miR-766-3p | RP11-142C4.6 |
| hsa-miR-27a-3p | LINC01123 |
| hsa-miR-1237-3p | RP11-90K6.1 |
| hsa-miR-185-5p | CTA-280A3.2 |
| hsa-miR-766-3p | LINC01002 |
| hsa-miR-765 | ST20-AS1 |
| hsa-miR-194-3p | FAM95B1 |
| hsa-miR-1200 | RP11-627G23.1 |
| hsa-miR-149-3p | CTA-315H11.2 |
| hsa-miR-125a-3p | LINC00686 |
| hsa-miR-149-3p | RP11-153F5.7 |
| hsa-miR-149-3p | LINC00173 |
| hsa-miR-139-5p | AC015849.16 |
| hsa-miR-185-5p | RP11-159D12.10 |
| hsa-miR-185-5p | LINC00265 |
| hsa-miR-423-5p | SRRM2-AS1 |
| hsa-miR-150-5p | AC015849.13 |
| hsa-miR-149-3p | TMEM191A |
| hsa-miR-1207-5p | LINC00969 |
| hsa-miR-423-5p | RP11-14P20.1 |
| hsa-miR-338-3p | AC015849.16 |
| hsa-miR-149-3p | RP11-186N15.3 |
| hsa-miR-129-5p | LINC00662 |
| hsa-miR-1207-5p | H19 |
| hsa-miR-1207-5p | RP5-1142A6.2 |
| hsa-miR-1200 | RP11-1129I3.1 |
| hsa-miR-423-5p | RP11-186N15.3 |
| hsa-miR-338-3p | SNHG14 |
| hsa-miR-20a-3p | LINC00906 |
| hsa-miR-539-5p | ZNF883 |
| hsa-miR-766-3p | CTD-2311B13.1 |
| hsa-miR-1226-3p | RP11-982M15.7 |
| hsa-miR-558 | CITF22-24E5.1 |
| hsa-miR-539-5p | CTC-435M10.10 |
| hsa-miR-150-5p | RP11-38M8.1 |
| hsa-miR-558 | RP11-503N18.1 |
| hsa-miR-136-5p | SPACA6P |
| hsa-miR-766-3p | CTC-338M12.9 |
| hsa-miR-129-5p | RP11-69I8.2 |
| hsa-miR-502-5p | AC010524.2 |
| hsa-miR-139-5p | RP11-231D20.2 |
| hsa-miR-513a-3p | RP11-474P2.6 |
| hsa-miR-767-5p | RP11-223P11.3 |
| hsa-miR-766-3p | AP001631.9 |
| hsa-miR-765 | AC005324.6 |
| hsa-miR-149-3p | CTD-3193O13.1 |
| hsa-miR-1200 | LINC00689 |
| hsa-miR-558 | RP11-638I8.1 |
| hsa-miR-1226-3p | AC093642.4 |
| hsa-miR-766-3p | TTN-AS1 |
| hsa-miR-34a-3p | CTD-2619J13.19 |
| hsa-miR-921 | RP11-326C3.14 |
| hsa-miR-34a-3p | GS1-279B7.1 |
| hsa-miR-767-5p | MCF2L-AS1 |
| hsa-miR-27a-3p | CTD-2281E23.1 |
| hsa-miR-21-3p | RP11-130C6.1 |
| hsa-miR-149-3p | MAFG-AS1 |
| hsa-miR-214-3p | CTC-242N15.1 |
| hsa-miR-129-5p | RP11-67K19.3 |
| hsa-miR-765 | ATP2A1-AS1 |
| hsa-miR-338-3p | CTD-3138B18.5 |
| hsa-miR-1207-5p | LINC01168 |
| hsa-miR-129-5p | REV3L-IT1 |
| hsa-miR-1237-3p | RP11-407A16.4 |
| hsa-miR-615-3p | RP11-689K5.3 |
| hsa-miR-1207-5p | AC000095.11 |
| hsa-miR-766-3p | CTD-3099C6.5 |
| hsa-miR-214-3p | NR2F1-AS1 |
| hsa-miR-149-3p | RP11-148K1.12 |
| hsa-miR-129-5p | RP5-1125A11.7 |
| hsa-miR-490-5p | CDR1-AS |
| hsa-miR-340-5p | LINC00869 |
| hsa-miR-338-3p | GRM7-AS1 |
| hsa-miR-214-3p | CTC-321K16.1 |
| hsa-miR-539-5p | SATB1-AS1 |
| hsa-miR-149-3p | RP11-430G17.3 |
| hsa-miR-129-5p | RP3-508I15.22 |
| hsa-miR-126-5p | RP11-517O13.1 |
| hsa-miR-149-3p | RP11-1348G14.8 |
| hsa-miR-539-5p | LINC01539 |
| hsa-miR-129-5p | SEPSECS-AS1 |
| hsa-miR-9-5p | RP11-397O4.1 |
| hsa-miR-125a-3p | RP11-982M15.8 |
| hsa-miR-149-3p | PAX8-AS1 |
| hsa-miR-125a-3p | LINC00689 |
| hsa-miR-766-3p | CTD-2008P7.1 |
| hsa-miR-513a-3p | LL22NC03-N64E9.1 |
| hsa-miR-185-5p | SSTR5-AS1 |
| hsa-miR-539-5p | RP11-598F7.3 |
| hsa-miR-129-5p | RP11-848P1.3 |
| hsa-miR-423-5p | RP11-394A14.2 |
| hsa-miR-129-5p | RP11-486O12.2 |
| hsa-miR-1226-3p | RP4-591C20.9 |
| hsa-miR-129-5p | RP1-283E3.8 |
| hsa-miR-28-3p | LINC00662 |
| hsa-miR-149-3p | RP11-630C16.2 |
| hsa-miR-766-3p | ABHD11-AS1 |
| hsa-miR-130b-5p | RP11-486P11.1 |
| hsa-miR-129-5p | RP11-189E14.3 |
| hsa-miR-513a-3p | AC009299.3 |
| hsa-miR-767-5p | RP4-539M6.22 |
| hsa-miR-125a-3p | CTD-2291D10.2 |
| hsa-miR-340-5p | RP11-374A4.1 |
| hsa-miR-1226-3p | HTR5A-AS1 |
| hsa-miR-27a-3p | AC078942.1 |
| hsa-miR-185-5p | RP11-458F8.4 |
| hsa-miR-539-5p | EGFLAM-AS3 |
| hsa-miR-1207-5p | MIRLET7BHG |
| hsa-miR-1237-3p | CTC-548K16.6 |
| hsa-miR-186-3p | RP11-368I7.4 |
| hsa-miR-149-3p | CTD-2369P2.8 |
| hsa-miR-765 | RP11-570L14.2 |
| hsa-miR-20a-3p | CTD-2066L21.2 |
| hsa-miR-214-3p | LINC01304 |
| hsa-miR-130b-5p | CTC-457E21.1 |
| hsa-miR-129-5p | RP4-794I6.4 |
| hsa-miR-922 | LA16c-306A4.2 |
| hsa-miR-423-5p | HP09025 |
| hsa-miR-766-3p | RP11-347H15.4 |
| hsa-miR-922 | RP4-539M6.22 |
